# ASAP1 activates the IQGAP1/CDC42 pathway to promote tumor progression and chemotherapy resistance in gastric cancer

**DOI:** 10.1038/s41419-023-05648-9

**Published:** 2023-02-15

**Authors:** Wangkai Xie, Zheng Han, Ziyi Zuo, Dong Xin, Hua Chen, Juanjuan Huang, Siyu Zhu, Han Lou, Zhiqiang Yu, Chenbin Chen, Sian Chen, Yuanbo Hu, Jingjing Huang, Fabiao Zhang, Zhonglin Ni, Xian Shen, Xiangyang Xue, Kezhi Lin

**Affiliations:** 1 Department of General Surgery, The Second Affiliated Hospital and Yuying Children’s Hospital of Wenzhou Medical University, Wenzhou, China; 2 Department of General Surgery, The First Affiliated Hospital of Wenzhou Medical University, Wenzhou, China; 3 Wenzhou Collaborative Innovation Center of Gastrointestinal Cancer in Basic Research and Precision Medicine, Wenzhou Key Laboratory of Cancer-related Pathogens and Immunity, Experiemtial Center of Basic Medicine, Department of Microbiology and Immunology, Institute of Molecular Virology and Immunology, School of Basic Medical Sciences, Wenzhou Medical University, Wenzhou, China; 4 Department of emergency, The Second Affiliated Hospital and Yuying Children’s Hospital of Wenzhou Medical University, Wenzhou, China; 5 Department of Pathology, The Second Affiliated Hospital and Yuying Children’s Hospital of Wenzhou Medical University, Wenzhou, China; 6 Key Laboratory of Minimally Invasive Techniques & Rapid Rehabilitation of Digestive System Tumor of Zhejiang Province, Department of Hepatic-biliary-pancreatic Surgery Taizhou Hospital of Zhejiang Province affiliated to Wenzhou Medical University, 317000 Zheiang Province Linhai, China

**Keywords:** Gastric cancer, Prognostic markers

## Abstract

Abnormal expression and remodeling of cytoskeletal regulatory proteins are important mechanisms for tumor development and chemotherapy resistance. This study systematically analyzed the relationship between differential expression of cytoskeleton genes and prognosis in gastric cancer (GC). We found the Arf GTP-activating protein ASAP1 plays a key role in cytoskeletal remodeling and prognosis in GC patients. Here we analyzed the expression level of ASAP1 in tissue microarrays carrying 564 GC tissues by immunohistochemistry. The results showed that ASAP1 expression was upregulated in GC cells and can be served as a predictor of poor prognosis. Moreover, ASAP1 promoted the proliferation, migration, and invasion of GC cells both in vitro and in vivo. We also demonstrated that ASAP1 inhibited the ubiquitin-mediated degradation of IQGAP1 and thus enhanced the activity of CDC42. The activated CDC42 upregulated the EGFR-MAPK pathway, thereby promoting the resistance to chemotherapy in GC. Taken together, our results revealed a novel mechanism by which ASAP1 acts in the progression and chemotherapy resistance in GC. This may provide an additional treatment option for patients with GC.

## Introduction

Gastric cancer (GC) is an aggressive and poorly understood malignancy [1]. It is the fifth most common type of cancer and the third most common cause of cancer deaths across the world. In 2018, more than one billion new cases and 783,000 deaths of patients with GC were reported [2]. The early stages of GC are clinically silent, and the treatment of advanced GC primarily depends on surgical resection, radiotherapy and chemotherapy [3]. Because of recurrence, metastasis and chemotherapy resistance, the prognosis of GC patients is dismal and the average 5-year survival rate is <20% [4]. Therefore, the mechanisms underlying metastasis and chemotherapy resistance in GC must be identified for the development of treatment solutions [5].

Tumor development and chemotherapy resistance may arise from abnormal expression and remodeling of cytoskeletal regulatory proteins [6]. Altered cellular motility is a hallmark of metastasis, which is a complex process requiring dramatic remodeling of the cytoskeleton [7, 8]. Previous studies have demonstrated that the epithelial-to-mesenchymal transition (EMT) as well as abnormal expression and remodeling of cytoskeletal regulatory proteins are important mechanisms for the invasion and chemotherapy resistance of GC cells [9–11]. In combination with supporting EMT, the Hippo signaling through YAP activation helps mediate the independence from oncogenic mitogen-activated protein kinase (MAPK) signaling and the resistance to MAPK inhibitor treatment [12]. Moreover, several studies have found that the activated RHOA–ROCK–myosin II signaling, which is required for cytoskeletal activity, dampens reactive oxygen species induction, supports DNA damage repair, and upregulates immunosuppressive PD-L1 in tumor cells, thereby increasing resistance to chemotherapy [6].

Elucidating the mechanisms of action of abnormal cytoskeletal regulatory proteins in GC is thus of vital significance for improving patient survival [13]. In this study, we systematically analyzed the relationship between differential expression of cytoskeleton genes and prognosis in gastric cancer. Adenosine diphosphate ribosylation factor guanylate kinase 1 (ASAP1, AMAP1, DDEF1, DEF1, or centaurin β4), which is the ADP-ribosylation factor (ARF) GTPase-activating protein, regulates cell movement and cytoskeletal remodeling [14–17]. ASAP1 contains multiple domains. The N-terminal BAR domain functions as a site for protein-protein binding, and both ankyrin and SH3 domains facilitate the interactions between ASAP1 and focal adhesion kinase (FAK), CD2AP, and Src [18–22]. The pleckstrin homology domain regulates the ARF state between GDP and GTP [23, 24]. The role of the ASAP1 in promoting malignant phenotypes has been found in a variety of cancer types, and its correlation with patient prognosis suggests its potential clinical value [25–29]. Studies have shown that ASAP1 is highly expressed in GC tissues, which is associated with patient survival [20]. However, the biological role of ASAP1 in GC and the molecular mechanisms underlying its influence on resistance to chemotherapeutic drugs remain unclear. Therefore, the aim of this study was to elucidate the contribution of ASAP1 in the dysregulation of cytoskeletal proteins to tumor progression and chemotherapy resistance in GC cells.

## Materials and methods

### Bioinformatics analysis

Differential expression analysis was performed using the DEseq2 package (https://github.com/mikelove/DESeq2) to find differentially expressed genes (DEGs) between normal and tumorous tissues across three Gene Expression Omnibus (GEO) datasets (GSE13911, GSE63089, and GSE65801). Next, univariate Cox analysis was applied to these genes in two GEO datasets (GSE66229 and GSE15460) to find survival-related DEGs. To construct a hybrid cytoskeleton-related risk scoring model, the GSE66229 and GSE15460 datasets were utilized, both of which have related clinicopathological factors as training and external validation datasets. To evaluate the prognostic ability of the model, the receiver operating characteristic (ROC) and Kaplan–Meier survival analyses were performed on the training and external validation cohorts.

### Clinical specimens

We obtained 53 frozen GC specimens and paired tumor-adjacent normal gastric tissues. These were used to extract total RNA and subjected to quantitative polymerase chain reaction (qPCR). We obtained 12 GC tissues which were cultured in 3D for drug sensitivity analysis. We also obtained 564 paraffin-embedded GC tissues and 81 adjacent non-cancerous tissues for ASAP1 immunohistochemistry (IHC). Informed consent was obtained from each patient. The entire study was approved by the Second Affiliated Hospital of Wenzhou Medical University (Wenzhou, China). The gastric adenocarcinoma and adjacent tissue samples were fixed with formalin (at least 10 cm from the negative edge) and subjected to histopathological analysis. According to the Joint Committee on Cancer Staging Manual, the demographic and clinicopathological characteristics of the patients included age, differentiation status, sex, serum carcinoembryonic antigen, Lauren type, levels of CA 72-4 and CA19-9, depth of invasion, lymph node metastasis, and tumor node metastasis (TNM) stages at the time of surgery.

### Cell transfections

The HA-tagged ASAP1 open reading frame DNA fragment was inserted into the BamHI and XhoI restriction sites of the pcDNA3.1 (+) vector. The Flag-labeled EGFR open reading frame DNA fragment was inserted into the BamHI and XhoI restriction sites of the pcDNA3.1(+) vector. The constructed plasmid was verified by sequencing. The expression of ASAP1-HA in GC cells was confirmed by HA tag. The plasmid was transfected into GC cells using a Lipofectamine 2000 reagent(Invitrogen, Carlsbad, CA, USA) according to the manufacturer’s instructions.

Two siRNA oligos against ASAP1 or IQGAP1 were designed and synthesized by RiboBio (Table S2. Guangzhou, China). GC cells were seeded at 2 × 10^5^ cells/well in 6-well plates and cultured for 24 h. The siRNAs were transfected into GC cells at a final concentration of 20 nmol using Lipofectamine 2000 reagent (Invitrogen). The single guide RNA (sgRNA) sequence used for the knock out of ASAP1 withCRISPR-Cas9 (sgASAP1: GTTCATCGCCGAGACCACCG) was inserted into plasmid pSpCas9(BB)-2A-Puro (PX459) purchased from Addgene(#48139). The plasmid was transfected into GC cell line SGC-7901 using a Lipofectamine 2000 reagent, and the cells were selected with 2 µg/ml puromycin (Sigma‐Aldrich, St. Louis, MO, USA).

### Immunohistochemistry

Paraffin-embedded tissue samples of 5-µm sections were subjected to IHC staining. Tissue sections were deparaffinized and rehydrated in graded ethanol solutions. Then, the deparaffinized tissue specimens were boiled in 10 mM citrate buffer (pH 6.0) for 100 min for antigen retrieval. Subsequently, the endogenous peroxidase activity was blocked by incubating with 3% H_2_O_2_ solution. The tissue sections were then incubated with the antibody in a humidified chamber at 37 °C for 2 h (Table S3). After washing thrice with phosphate buffered saline (PBS), a Dako EnVision FLEX detection system (Dako, Carpinteria, CA, USA) was used for visualization, according to the manufacturer’s instructions. The sections were counterstained with hematoxylin, dehydrated, and sealed with neutral gum. IHC staining was evaluated by experienced pathologists using a semi-quantitative scoring system based on the intensity of staining and the percent of positively-stained cells. The staining intensity of specimens was scored as 0 (negative), 1 (weak), 2 (moderate), and 3 (strong). The positive expression was scored as 0 (0%), 1 (<25%), 2 (25–50%), 3 (50–75%), and 4 (>75%) based on the percentage of positive stained cells. The final staining scores for all samples were obtained by multiplying the staining intensity.

### RNA isolation and qPCR

Total RNA was extracted from cells and GC tissues using TRIZOL reagent. After its concentration was determined two-step reverse transcription (TOYOBO, Tokyo, Japan, fsq301) was used for cDNA synthesis followed by qPCR. Sequence of ASAP1 and housekeeping gene GAPDH primers was listed in Table S1. ASAP1 mRNA data were normalized to the GAPDH housekeeping gene.

### Cell culture

Two GC cell lines, MGC‐803 and SGC-7901, were purchased from the Cell Bank of the Chinese Academy of Sciences (Shanghai, China). These cell lines were grown in Dulbecco’s modified Eagle’s medium (DMEM, Gibco, Grand Island, NY, USA) supplemented with 10% fetal bovine serum (FBS; Gibco) in a humidified chamber at 37 °C and 5% CO2.

### CCK-8, cell colony formation, and EdU assays

For the Cell Counting Kit-8(CCK-8) assay, GC cells (5000 cells per well) seeded into 96-well plates. After transfection for 24 h, 48 h and 72 h, 10 μl of CCK-8 reagent (Dojindo, Kumamoto, Japan) reagent was added into each well and incubated for 3 h at 37 °C. The absorbance was measured at a wavelength of 450 nm. All experiments were carried out in triplicate.

For the cell colony formation assay, GC cells (500 cells per well) were seeded in 6‑well plates at 37 °C for 2 weeks, the medium was changed every 3 days. Then, cell colonies were fixed with 4% paraformaldehyde for 15 min and stained with 0.01% crystal for 15 min at 25 °C. Cell colonies containing >20 cells were counted. All experiments were carried out in triplicate.

For the 5-Ethynyl-2-deoxyuridine (EdU) assay, GC cells (8,000 cells per well) were seeded into 96-well plates. After transfection 24 h, 50 μM of EdU (Cell Light EdU DNA imaging Kit, RiboBio) reagent were added into each well and incubated at 37 °C for 2 h. GC cells were fixed with 4% paraformaldehyde for 30 min, permeabilized with 0.1% Triton X-100–PBS for 10 min, stained with EdU Apollo®567 and Hoechst for 30 min at 25 °C. The proportion of EdU-positive cells was visualized by fluorescence. All experiments were carried out in triplicate.

### Cell cycle and apoptosis assays

For the cell cycle assays, GC cells (5 × 10^5^ cells per well) were seeded into 6-well plates. Cells were collected 48 h after transfection, fixed with 75% ethanol for 1 h at −20 °C, suspended to 1 million cells/mL and treated with propidium iodide/RNase staining solution (BD Pharmingen, San Jose, CA, USA) for 15 min at 25 °C. BD flow cytometry was used for detection. All experiments were carried out in triplicate.

For the cell apoptosis assays, GC cells (5 × 10^5^ cells per well) were seeded into 6-well plates. Cells were collected 48 h after transfection, closed with 5% BSA for 15 min at −20 °C, suspended to 1 million cells/mL and treated with Annexin-V-FITC and propidium iodide (BD Pharmingen) for 15 min at 25 °C, BD flow cytometry was used for detection. All experiments were carried out in triplicate.

### Transwell migration/invasion assays and Wound healing assays

The polycarbonate membrane in the Transwell chambers were coated with Matrigel (Corning, NY, USA). We transferred 1 × 10^5^ cells in serum-free medium into the top chamber and added medium with serum in the bottom chamber, and incubated at 37 °C for 24 h(SGC-7901) or 10 h(MGC-803). Then, we removed the non-invading cells on the top side of the membrane by scrubbing, fixed the migrating or invading cells at the bottom side of the membrane with 4% paraformaldehyde (PFA), and stained with 0.5% crystal violet. The number of cells were counted under a microscope (Leica, London, UK) from five randomly chosen fields per well to determine the number of cells in each group.

For wound healing assays, GC cells (5 × 10^5^ cells per well) were seeded into 6-well plates. After transfection, a scratch wound was made using a sterile 10 μL pipette tip in a monolayer of GC cells. The scratch wound was imaged at the 0, 12 and 24 h time points using microscope at the same location of 6-well plates. Wound healing rate was measured by mean distance between two edges of cell free area.

### In vivo experiment

Four-week-old athymic female BALB/c nu/nu mice were purchased from the Animal Experiment Center, Hangzhou Medical College (Hangzhou, China). All animal experiments complied with Wenzhou University’s Policy on the Care and Use of Laboratory Animals.

Mice were randomly divided into two experimental groups (*n* = 5), the SGC-7901-WT and ASAP1^+/−^ SGC-7901 (1 × 10^7^ per mice) were injected subcutaneously into the right backside of the nude mice. Oxaliplatin(Oxa) or control saline was intraperitoneally injected daily. Tumor volumes were determined by measuring the lengths (l) and widths (w) every 5 days. The mice were sacrificed, tumor tissues were removed and photographed. The tissues were paraffin-embedded and cut in 5-µm sections for immunohistochemical analysis.

The GC cell lines SGC-7901-WT and ASAP1^+/−^ SGC-7901 cells were infected with lentivirus expressing the luciferase reporter gene (OBiO Technology, Shanghai, China, # H9911) to construct SGC-7901-WT-Luc and ASAP1^+/−^ SGC-7901-Luc, which can continuously express the luciferase gene. Mice were divided into two experimental groups (*n* = 5) randomly, the SGC-7901-WT-Luc and ASAP1^+/−^ SGC-7901-Luc cells (1 × 10^6^ per mice) were injected into the tail veins of nude mice to construct lung metastasis models. Thirty minutes after the injection, each mouse was intraperitoneally injected with 15 mg/kg luciferin (BioGold). Five minutes later, the photon number of each mouse was detected using a small-animal imaging system (IVIS Lumina III, PerkinElmer) to confirm the same amount of cell injection in both groups. A month after cell injection, the photon number of each mouse was determined to compare the differences in lung metastases between the two cell lines.

### GTPases activity analysis

GC cells (50000 cells per well) were seeded into 6-well plates. After transfection 24 h cells were lysed after transfection 48 h and supernatants were collected. The levels of activated Rho GTPases were detection with The Cdc42/RhoA/Rac1 G-LISA kit (Cytoskeleton. Denver, CO, USA). The absorbance was measured at a wavelength of 490 nm. All experiments were carried out in triplicate.

### Immunofluorescence staining

GC cells (50,000 cells per well) were seeded into 6-well plates. After transfection 24 h cells were fixed with 4% paraformaldehyde at 25 °C for 10 min and permeabilized with 0.1% Triton X-100 at 25 °C for 15 min. Then, cells were blocked with 10% goat serum (Beyotime, Haimen, China) in PBS at 25 °C for 30 min. The cells were incubated with the antibody at 4 °C overnight, incubated with the Immunofluorescence staining (IF) secondary antibody at 37 °C for 1 h and then incubated with DAPI (Beyotime) at 25 °C for 3 min (Table S3). The coverslips were mounted, and the signals were visualized under a fluorescent microscope (Carl Zeiss, Jena, Germany).

### Western blotting and co‐immunoprecipitation

We extracted total proteins from human GC tissues and cell lines using the RIPA lysis buffer (Beyotime) supplemented with cocktail (Sigma-Aldrich, St. Louis, MO, USA). Then, equal amounts of the protein lysates were separated on a 10% SDS PAGE. The resolved proteins were transferred onto polyvinylidene fluoride (PVDF) membranes (Bio-Rad, Hercules, CA, USA) and blocked with 5% non-fat milk for 2 h at 25 °C. Then, the membrane was incubated with antibodies overnight at 4 °C, incubated with horseradish peroxidase-labeled second antibody for 2 h and visualized using a Bio-Rad imaging system.

The transfected cells were lysed with NP-40 containing a protease inhibitor cocktail (MCE). Protein supernatant was collected and mixed with antibody, and incubated overnight at °C, antibody quantities are shown in Table S3. After the supernatant was removed, the beads were washed with PBST three times and incubated with diluted elution buffer at 95 °C for 5 min. Finally, the pulled down protein complexes were analyzed by SDS–PAGE/immunoblotting analysis.

### Fluorouracil and oxaliplatin sensitivity prediction

To explore if the expression of ASAP1 could affect the drug sensitivity of fluorouracil and oxaliplatin, a ridge regression-based algorithm built in the “oncoPredict” R package was introduced. First, the TCGA-STAD TPM expression matrix with Log2(x + 1) transform was used as the predicting cohort. All patients with a follow-up time of fewer than 5 days were excluded, and the remaining patients were further separated into ASAP1 high- and low-expression groups using the X-tile (Version 3.6.1) software. To accurately predict drug sensitivity, we used the built-in CTRPv2 cohort data as the training cohort, and AGS, HGC27, MKN1, MKN45, MKN7, and MKN74 were used as the target cell lines. After performing the “calcPhenotype” function, a matrix with predicted drug sensitivity was acquired, and box plots with t-tests were used to visualize the imputed drug sensitivity.

### 3D in vitro model of human GC

To generate a 3D in vitro model of GC, harvested tissues were washed with PBS and cut into pieces of 0.8–1.2 mm in diameter. Tumors were transferred into 6-well plates (4–6 tissues per well, with 6 wells for each group). Each well was maintained with 1 mL of culture medium. The full medium was supplemented with advanced DMEM/F12 (Gibco,), B-27 (50×, Gibco), and antibiotic-antimycotic solution (Gibco). The tissues were cultured in a humidified incubator at 37 °C with 5% CO_2_.

### Statistical analysis

Statistical analysis and mapping were performed using SPSS Statistics (version 23.0; IBM SPSS, Chicago, IL), GraphPad Prism 7 (GraphPad Software, CA, USA), and the R&R studio (R&R studio) software. Analysis of variance and independent-sample t-tests were performed to assess differences between the groups. TCGA, GEO, and immunohistochemical expression data were divided into high and low groups using X-tile software. The prognostic significance of ASAP1 was evaluated using Kaplan–Meier plots. A Cox regression model was used to analyze independent risk factors for survival. Statistical significance was set at *P* < 0.05.

## Results

### Cytoskeleton-related risk scoring model predicts survival outcomes of patients with GC and exhibits an ability of highly prognostic prediction

**Fig. 1:**
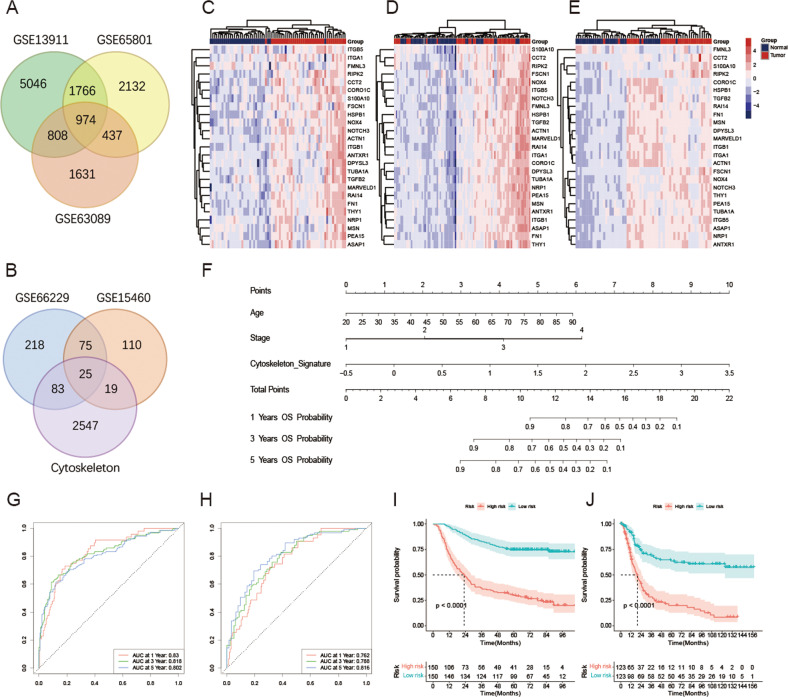
Extraction of cytoskeleton-related gene signature and construction of cytoskeleton-related risk scoring model. **A** Co-differential expressed genes in GSE13911, GSE63089, and GSE65801 datasets. **B** The intersection of survival-associated differential-expressed cytoskeleton-related gene signatures in GSE66229 and GSE15460 datasets. Expression profile of 25 survival-associated cytoskeleton-related gene signatures in GSE13911 (**C**), GSE63089 (**D**), and GSE65801 (**E**) datasets. **F** Cytoskeleton-related risk scoring model constructed for predicting the overall survival of patients with GC. Receiver Operator Characteristic (ROC) curve of cytoskeleton-related risk scoring model predicting the 1-, 3-, and 5-year overall survival of GC patients in the training (**G**) and validation (**H**) cohort. Kaplan–Meier analysis for GC patients between high- and low- cytoskeleton-related risk groups in the training (**I**) and validation (**J**) cohort. ns-not significant; *p < 0.05; **p < 0.01; ***p < 0.001.

A total of 974 co-differentially expressed genes were obtained by using the DEseq2 algorithm from GSE13911, GSE63089, and GSE65801 datasets (Fig. 1A). After a univariate Cox analysis of the GSE66229 and GSE15460 datasets, 100 genes showed a significant association with prognosis; among them, 25 genes were associated with the cytoskeleton (Fig. 1B). The heatmap demonstrates that the expression profile of these 25 cytoskeleton-related genes were associated with survival and differentially expressed between tumor and normal biotypes in GSE13911 (Fig. 1C), GSE63089 (Fig. 1D), and GSE65801 (Fig. 1E), respectively.

The clinicopathological factors and cytoskeleton-related gene signature composed of the 25 genes described above were utilized to construct the cytoskeleton-related risk scoring model. After multivariate Cox analysis, the age, AJCC stage, and cytoskeleton-related gene signature were identified as independent prognostic factors, and thus a hybrid model was constructed (Fig. 1F). ROC and Kaplan–Meier survival analyses were performed to examine the prognostic ability of the model. The results revealed that the cytoskeleton-related risk scoring model has an ability of favorable prognostic prediction. The 1-, 3-, and 5-year areas under the ROC curves (AUCs) of this model were 0.830, 0.818, and 0.802 in the training cohort, respectively (Fig. 1G), while the values for the external validation cohort (Fig. 1H) were 0.762, 0.788, and 0.816, respectively. Furthermore, the Kaplan–Meier survival analysis also showed distinct overall survival conditions between the high- and low-risk groups in both the training (Fig. 1I) and external validation cohorts (Fig. 1J).

### ASAP1 was highly expressed in GC tissues and associated with a poor prognosis

**Fig. 2:**
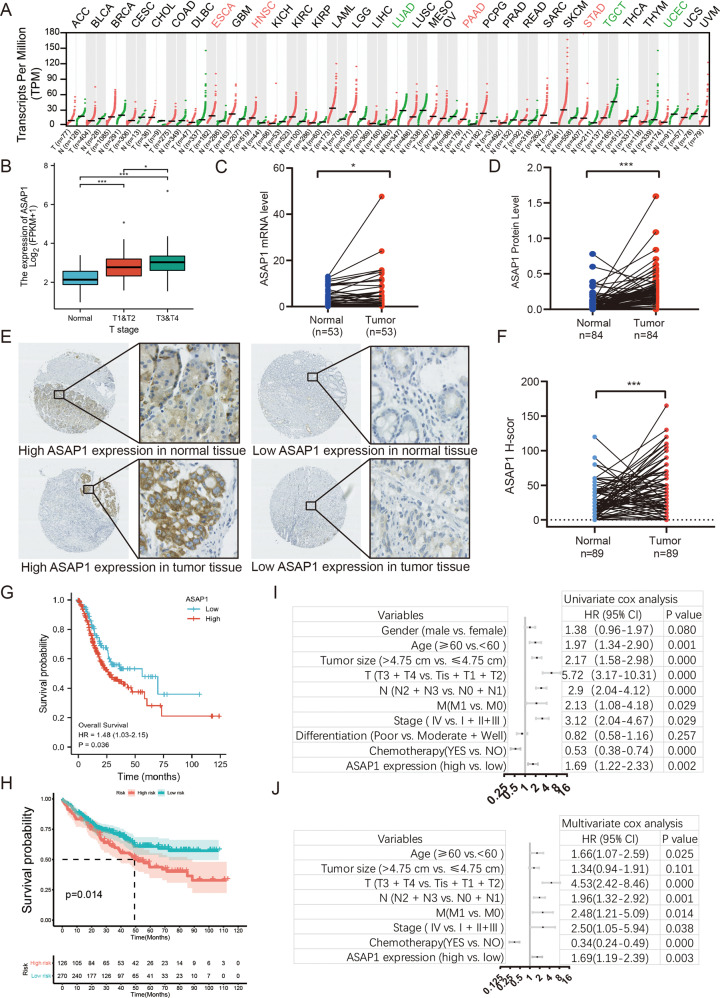
Clinical significance of ASAP1 expression in GC. **A** The histogram shows the ASAP1 expression in 27 kinds of human normal tissues. **B** The expression of ASAP1 in TCGA STAD in different T stages patients. **C** The expression levels of ASAP1 in 53 paired gastric tumor tissues and adjacent normal tissues were detected by real-time fluorescence quantitative PCR. **D** The expression levels of ASAP1 in 84 paired gastric tumor tissues and adjacent normal tissues detected by protein profiling. **E** Representative IHC images of ASAP1 staining in the GC and normal tissue samples, Bar in 40× and 400×. **F** Compared ASAP1 scores in 89 paired gastric tumor tissues and adjacent normal tissues. **G** Kaplan–Meier OS curves of patients with GC according to ASAP1 mRNA expression in TCGA dataset. **H** Kaplan–Meier OS curves of 551 patients with GC according to ASAP1 scores. **I**, **J** Indicated clinicopathologic features were brought into univariable risk factor analysis (**I**) and features associated with prognosis were further brought into multivariable risk factor analysis using Cox regression model (**J**). ns not significant; *p < 0.05; **p < 0.01; ***p < 0.001.

According to the TCGA data, ASAP1 is expressed at different levels in various types of cancer. ASAP1 was highly expressed in GC (STAD) (Fig. 2A). The ASAP1 expression increased with an increase in the GC T-stage and pathological stage (Fig. 2B). We detected the expression of ASAP1 mRNA in 53 paired GC and tumor-adjacent normal gastric tissues. We found that ASAP1 was highly expressed in GC tissues (Fig. 2C). We further downloaded the protein profile data of 84 pairs of GC patients and analyzed the expression of ASAP1 [30]. The results showed that ASAP1 was highly expressed in GC tissues (Fig. 2D). To investigate the patterns of ASAP1 expression and its clinical implications in patients with GC, we performed IHC staining to detect ASAP1 expression, using our archived GC tissue microarray. The results showed that ASAP1 was expressed mainly in the membrane of cancer cells and partially in the cytoplasm (Fig. 2E). The expression of ASAP1 was higher in tumor tissues than in the adjacent normal tissues (Fig. 2F). Based on the different H-scores obtained from IHC, we divided these patients into ASAP1 low (*n* = 107) and high expression (*n* = 457) groups. According to age (*P* = 0.0080), T-stage (*P* = 0.021), TNM stage (*P* = 0.037), there was a statistically significant difference between the patients with high and low ASAP1 expression, regardless of sex, N stage, differentiation, and distant metastasis (Table 1). These results suggest that ASAP1 might act as a common oncogene that participates in cancer progression. Moreover, TCGA-STAD patients were divided into two groups with high and low ASAP1 expression. Kaplan–Meier survival analysis revealed that patients with high expression of ASAP1 had poor prognosis (*P* = 0.036, Fig. 2G) and lower overall survival (OS) than those with low ASAP1 expression (*P* = 0.012, Fig. 2H). Furthermore, multivariate analysis revealed that ASAP1 expression was an independent prognostic factor for overall (Hazard Ratio=1.48, *P* = 0.036, Fig. 2I). Taken together, these results suggest that ASAP1 is highly expressed in GC tissues and associated with poor prognosis in patients with GC.

**Table 1:** Clinicopathological features and ASAP1 in GC.

Clinicopathological features	All patients (*n* = 547)	Low (*n* = 107, 19.0%)	High (*n* = 457, 81.0%)	*P*-value
Gender				0.141
Male	410	274	136	
Female	137	99	38	
Age^b^				0.008^a^
<60	178	134	44	
≥60	369	239	130	
Tumor size^b^				0.189
<4 cm	340	237	103	
≥4 cm	207	136	71	
Invasion depth				0.021^a^
T1 + T2	152	114	38	
T3 + T4	395	259	136	
lymphatic metastasis				0.232
N0 + N1	253	177	76	
N2 + N3	294	196	98	
Distant metastases				0.292
No	512	357	164	
Yes	26	16	10	
TNM classification				0.037^a^
I + II	181	133	48	
/ III + IV	366	240	126	
Differentiation				0.245^a^
Moderat + Well	319	215	104	
Poor	198	140	58	

^a^Statistically significant (*P* < 0.05).

### ASAP1 promotes GC cell proliferation in vitro and xenograft tumor growth in vivo

**Fig. 3:**
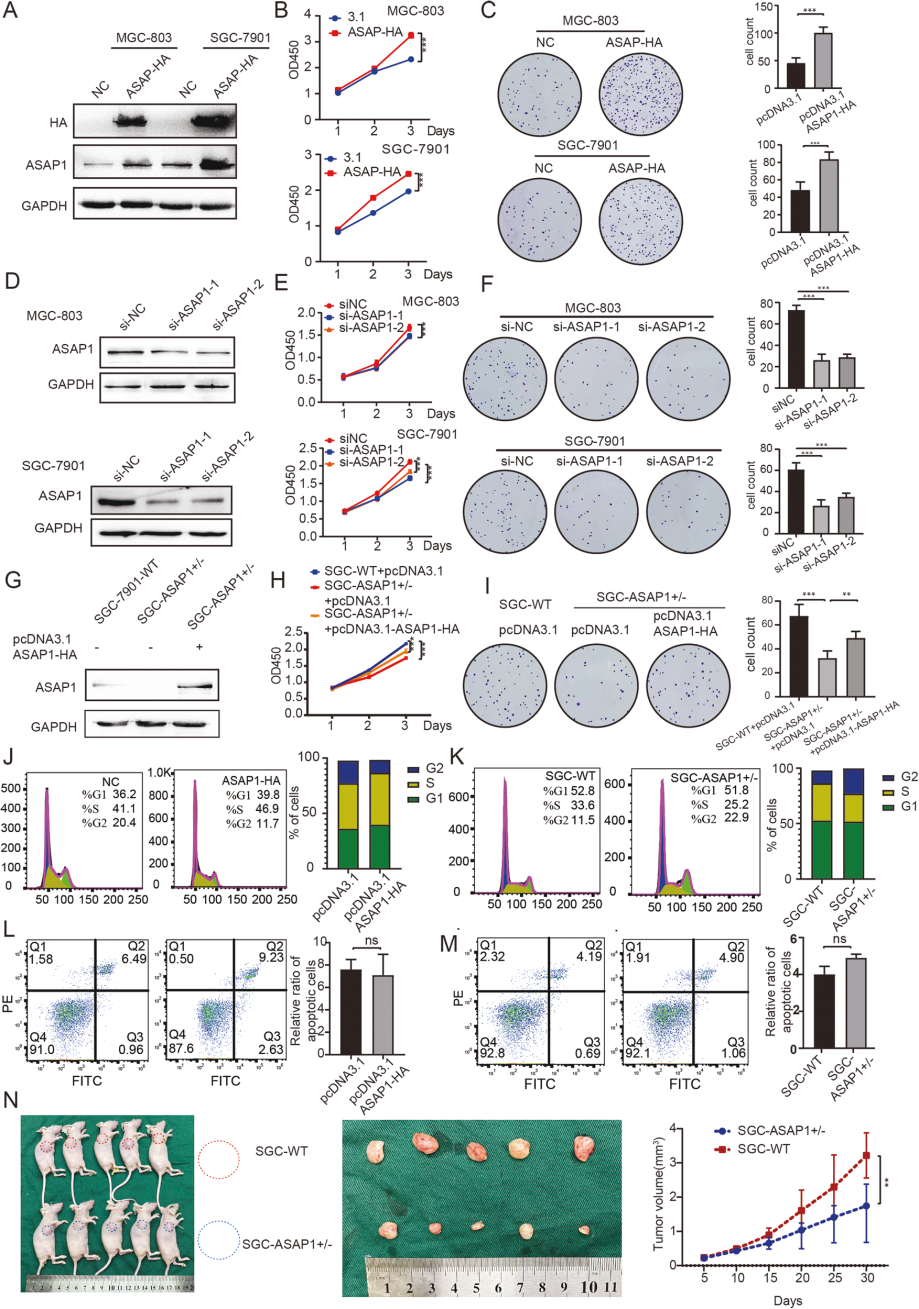
ASAP1 promotes GC cell proliferation in vitro and xenograft tumor growth in vivo. **A**, **D**, **G** Western blot analysis of ASAP1 of MGC-803 and SGC-7901 cells expressing ASAP1-HA, depleted of ASAP1 by siRNA or partially knocked out of ASAP1 by CRISPR-Cas9. **B**, **E**, **H** CCK-8 assays with MGC-803 and SGC-7901 cells expressing ASAP1-HA, depleted of ASAP1 by siRNA or partially knocked out of ASAP1 by CRISPR-Cas9. **C**, **F**, **I** Representative images from colony formation with MGC-803 and SGC-7901 cells expressing ASAP1-HA, depleted of ASAP1 or partially knocked out of ASAP1. **J**, **K** Cell cycle assay of SGC-7901 cells expressing ASAP1-HA or partially knocked out of ASAP1. **L**, **M** Apoptosis assay of SGC-7901 cells expressing ASAP1-HA or partially knocked out of ASAP1. **N** In vivo analysis using a tumor xenograft model. The growth curve of xenograft tumors from WT (*n* = 5) and ASAP1 ± (*n* = 5) SGC-7901 cells. ns not significant; *p < 0.05; **p < 0.01; ***p < 0.001.

To explore why the high expression of ASAP1 leads to a poor prognosis, we examined the proliferation and tumorigenic effect of ASAP1 on GC cells by overexpressing ASAP1 in both SGC-7901 and MGC-803 cells. We constructed a ASAP1-HA-tagged expression plasmid and transfected it into these cells. Western blotting results demonstrated that ASAP1-HA protein was highly expressed. (Fig. 3A). Immunofluorescence analysis further confirmed the expression of ASAP1-HA (Fig. S1A). The CCK-8 and colony formation assays showed that overexpression of ASAP1 resulted in an increased proliferation and viability of both SGC-7901 and MGC-803 cells (Fig. 3B, C). In contrast, depletion of ASAP1 by siRNA resulted in a marked decrease in cell proliferation and viability, compared to those in the control cells (Fig. 3D–F). In addition, heterozygous knockout of ASAP1 in SGC-7901 cells (ASAP1^+/−^) by CRISPR/Cas9 inhibited the cell proliferation. Notably, reintroduction of ASAP1 into ASAP1^+/−^ cells restored the cell proliferation (Figs. 3G–I and S1B). EdU assays with ASAP1 overexpressed cells increased proliferation and viability, and heterozygous knockout cells decreased in cell proliferation and viability (Fig. S2A).

We examined the distribution of cell cycle via flow cytometry to investigate the possible mechanism by which ASAP1 promotes the proliferation of GC cells. The results showed that the population of cells in the *G2* phase of the cell cycle was increased in ASAP1^+/−^ SGC-7901 cells, whereas the population of cells in the *G2* phase was significantly decreased in SGC-7901 cells overexpressing ASAP1 that promoted the progression of cell cycle (Fig. 3J, K). These results suggest that ASAP1 may promote cell proliferation by facilitating the passage of tumor cells from the *G2* to *M* phases. However, apoptosis analysis revealed that changing the expression of ASAP1 had no effect on apoptosis of SGC-7901 cells (Fig. 3L, M).

Next, we examined the influence of ASAP1 expression on the tumorigenic phenotype and tumor growth in vivo. The established mouse xenograft model was injected with wildtype (WT) (blue) or ASAP1^+/−^ SGC-7901 cells (red), and then tumor growth was monitored. The heterozygous knockout of ASAP1 significantly reduced the tumor volume in mice inoculated with SGC-7901 cells (Fig. 3N), Fig. S4A shows the results of hematoxylin-eosin (HE) staining and immunohistochemistry of the subcutaneous tumor. The results of the xenograft assay demonstrated that the SGC-7901 xenograft tumor growth was significantly reduced when ASAP1 was knocked down in SGC-7901 cells.

### ASAP1 promotes GC cell migration and invasion in vitro and in vivo

**Fig. 4:**
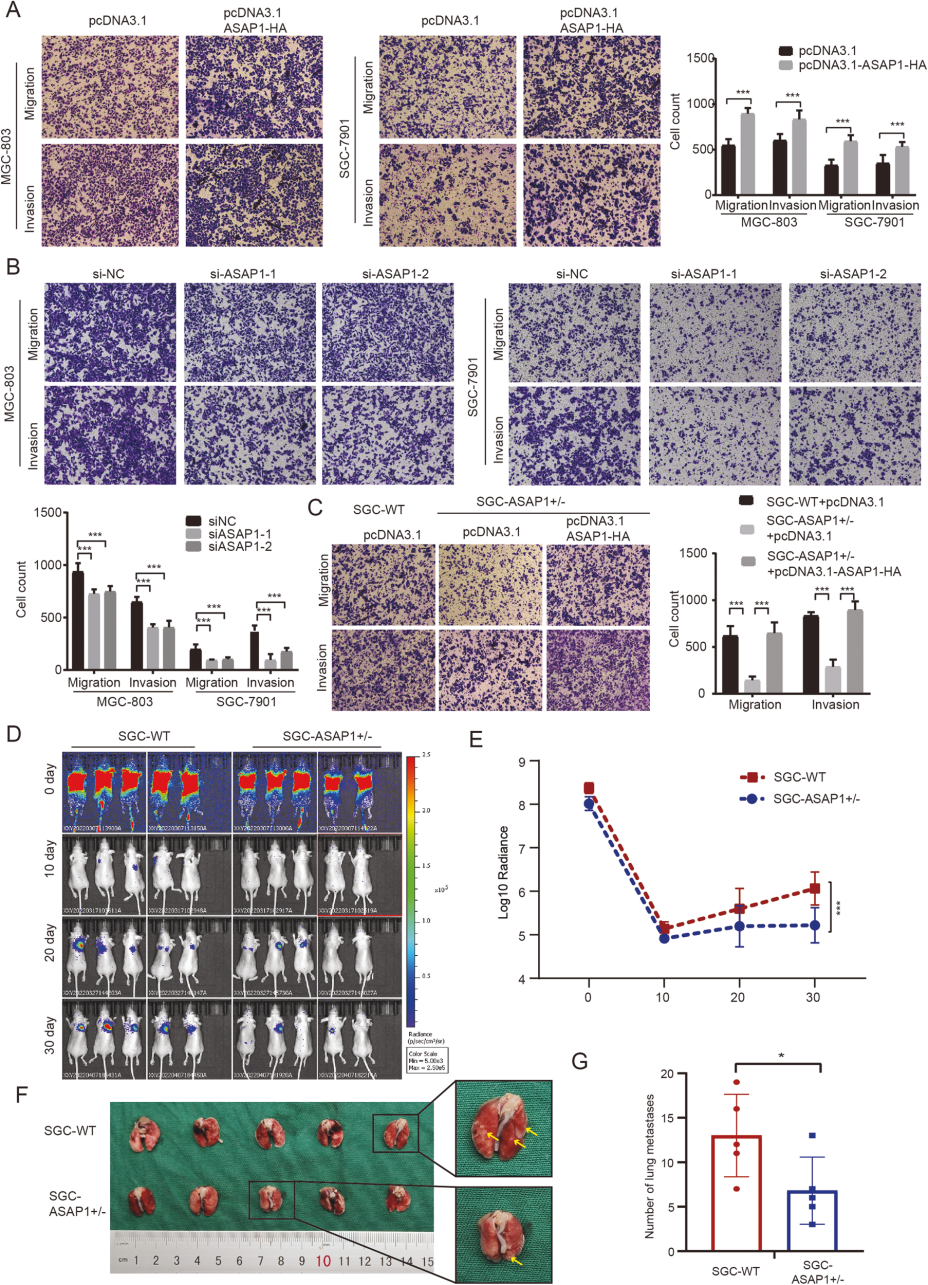
ASAP1 promote GC cell migration and invasion in vitro and in vivo. **A**, **B**, **C** Representative images from transwell assays with MGC-803 and SGC-7901 cells expressing ASAP1-HA, depleted of ASAP1 or partially knocked out of ASAP1. The numbers of migrating and invading cells are presented in the right panel. Scale bar,100 μm. **D**, **E** SGC-7901-WT-Luc and ASAP1^+/−^ SGC-7901-Luc were injected into tail veins of nude mice to construct lung metastasis models, respectively, 30 days after injection, metastasis of GC cells in lungs of nude mice was evaluated and quantified. **F**, **G** Specimen graphs of lung metastases and statistical graphs of the number of tumors metastasizing in the lungs ns-not significant; *P < 0.05, **P < 0.01, and ***P < 0.001.

Transwell migration, invasion, and scratch wound healing assays were performed to examine the effects of ASAP1 on the migration and invasion of GC cells in vitro. The transient expression of ASAP1-HA in both SGC-7901 and MGC-803 GC cells by transfection with pcDNA3.1-ASAP1-HA plasmid significantly enhanced cell migration and invasion (Fig. 4A). In contrast, depletion of ASAP1 by siRNA significantly inhibited the migration and invasion of both GC cell lines (Fig. 4B). These results were further confirmed by the wound scratch analysis (Fig. S2B). Moreover, Transwell analysis of ASAP1^+/−^ cells showed that heterozygous knockout of ASAP1 gene inhibited the migration and invasion of GC cells, and the reintroduction of ASAP1 into ASAP1^+/−^ cells restored the cell migration and invasion (Fig. 4C). To explore the specific role of ASAP1 in promoting the migration and invasion of GC cells, we depleted of ASAP1 using siRNA and detected the expression levels of E-cadherin (an epithelial marker) and N-cadherin (a mesenchymal marker). The results showed that the E-cadherin expression increased, whereas the N-cadherin expression decreased after the knockdown of ASAP1 (Fig. S3A).

To monitor the metastasis of tumor cells promoted by ASAP1 in vivo, lung metastasis models were constructed by injecting wildtype (WT) and ASAP1^+/−^ SGC-7901-Luc cells into the tail veins of nude mice, respectively. We found that, on the 30^th^ day after injection, SGC-7901-WT-Luc cells could form obvious lung metastases, whereas ASAP1^+/−^ SGC-7901-luc cells could hardly form metastases (Fig. 4D). The mice were sacrificed at the end of the 30th day, and then the tumors were excised and the number of tumors with metastasis in the lung was counted (Fig. 4E). Figure S4B shows the results of hematoxylin-eosin (HE) staining and immunohistochemistry of the tumors with metastasis. The results showed that the tumor tissues from ASAP1^+/−^ cells-injected mice were significantly reduced, compared to those from the mice injected with SGC-7901 WT cells (Fig. 4E).

### ASAP1 mediates cytoskeleton assembly by regulating CDC42 expression in GC cells

**Fig. 5:**
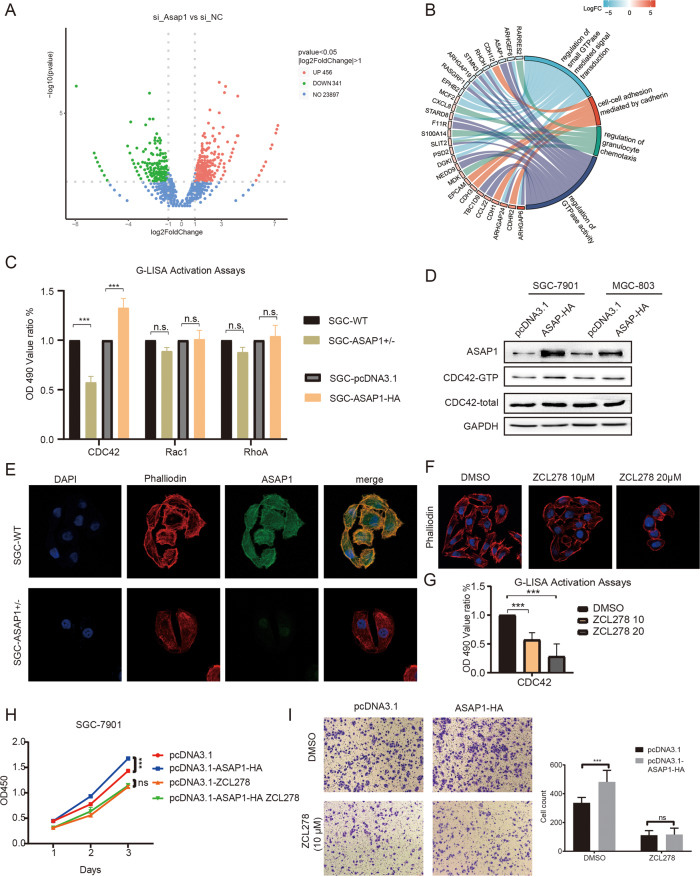
ASAP1 mediates cytoskeleton assembly by regulating CDC42 expression in GC cells. **A** The RNA-seq of ASAP1-depletedGC cell and a volcano plot showing the differential genes. **B** KEGG pathway analysis showing enrichment of differential gene. **C** The whole cell lysates were extracted from SGC-7901 cells expressing ASAP1-HA or from ASAP1^+/−^ SG-7901 cells to measure CDC42, RAC1, RALA and RhoA GTPase activity by G-LISA activation assay. **D** The whole cell lysates prepared from either SGC-7901 or MGC803 cell expressing ASAP1-HA were used for CDC42 activation assay followed by western blotting. **E** Combined phalloidin staining for F-actin (red) and ASAP1 (green) immunofluorescence. Nuclei were counterstained with DAPI (blue). In ASAP1^+/−^ SGC-7901 cells, the filamentous pseudopodia disappeared and cytoskeleton depolymerized. **F** SGC-7901 cells were treated with CDC42 inhibitor ZCL278 and combined phalloidin staining for F-actin (red). The filamentous pseudopodia disappeared and cytoskeleton depolymerized. **G** SGC-7901 cells were treated with CDC42 inhibitor ZCL278 and the whole cell lysates were extracted to measure CDC42 GTPase activity by G-LISA activation assay. **H** The proliferation of SGC-7901 cells transfected with pcDNA3.1, SGC-7901 cells transfected with pcDNA3.1-ASAP1-HA, SGC-7901 cells treated with ZCL278 or SGC-7901 cells transfected with pcDNA3.1-ASAP1-HA followed by treated with ZCL278 was determined by CCK-8. **I** The migration of various types of cells described in **H** was determined by Transwell assays.

Pathway analysis of the differentially expressed genes that downregulated ASAP1 expression showed that ASAP1 was closely related to the GTPase signaling pathway (Fig. 5A, B). Next, we assessed the activities of the GTPase family proteins (CDC42, Rac1, and RhoA) by using a G-LISA assay that specifically detects the active form (GTP-bound). The results showed that only activated CDC42 was diminished when ASAP1 was knocked down (Fig. 5C). Western blotting results showed that expression of ASAP1-HA in both SGC-7901 and MGC-803 cells resulted in an increased level of CDC42-GTP protein, whereas the total CDC42 protein remained unchanged (Fig. 5D). CDC42 is mainly involved in cytoskeletal recombination and participates in cell pseudopodia formation. We then examined the formation of filamentous pseudopodia in ASAP^+/−^ SGC-7901 cells by using an IF assay. The results showed that the number of filamentous pseudopods in ASAP1^+/−^ cells was significantly reduced, compared to that in the control cells (Fig. 5E). Notably, when SGC-7901 WT cells were treated with an inhibitor of CDC42, ZCL278, the number of filamentous pseudopods decreased with increasing ZCL278 concentrations (Fig. 5F, G). Overall, ASAP1 induced CDC42 activation in GC cells. The knockdown of ASAP1 or reduction in CDC42 activity can lead to a decrease in the number of filamentous pseudopods.

To determine whether the oncogenic function of ASAP1 depends on the activity of CDC42, we expressed ASAP1-HA and inhibited CDC42 activity simultaneously in SGC-7901 cells. The results showed that ASAP1-HA expression could not rescue the proliferation and metastasis of GC cells inhibited by ZCL278, while ZCL278 completely blocked the proliferation and metastatic-promoting effect of ASAP1 (Fig. 5H, I). These results indicated that the progression of GC promoted by ASAP1 is entirely dependent on CDC42 activity.

### ASAP1 enhances CDC42 activity by interacting with and inhibiting ubiquitin-mediated degradation of IQGAP1

**Fig. 6:**
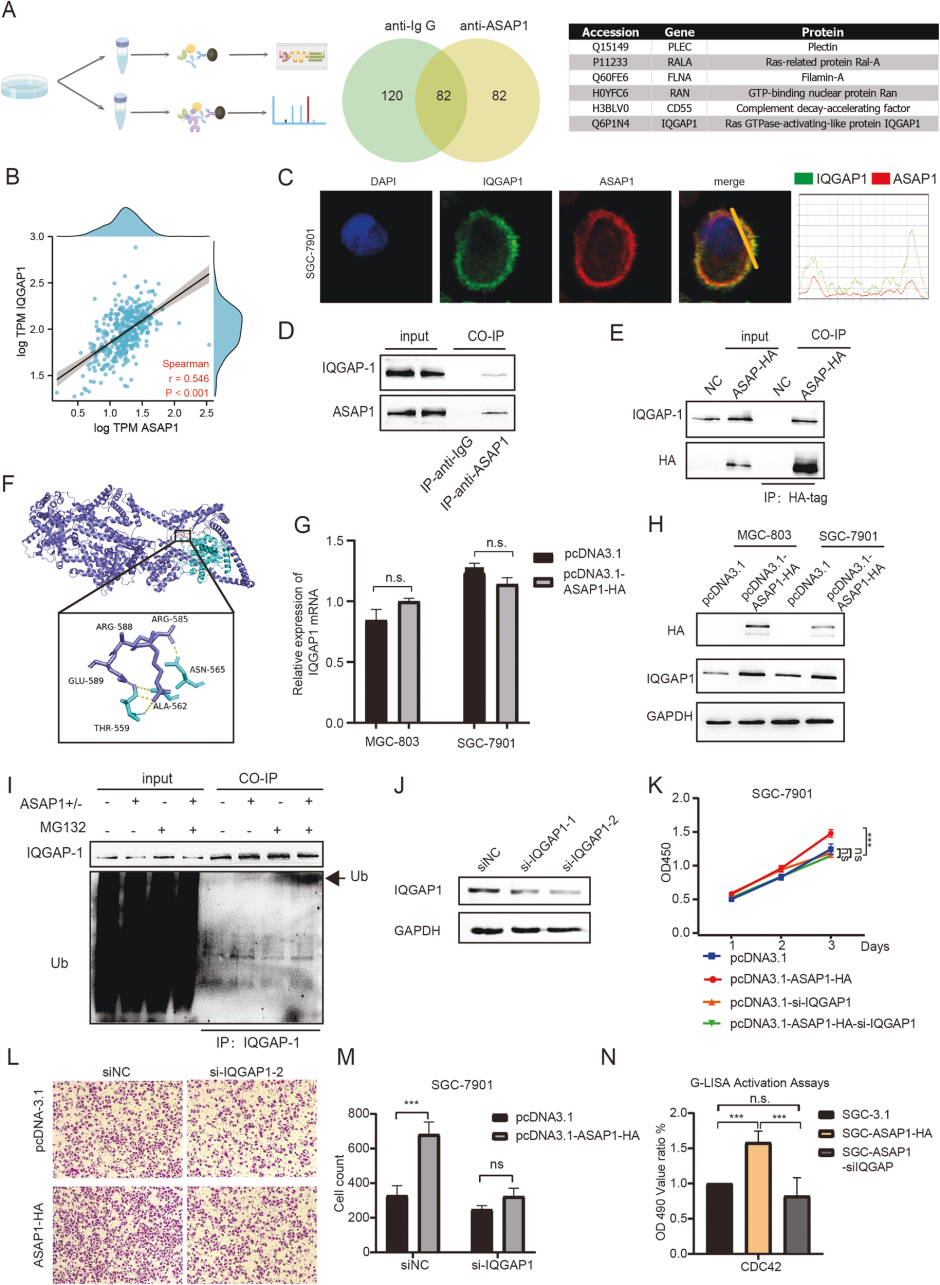
ASAP1 enhanced CDC42 by interacting with and inhibiting ubiquitin-mediated degradation of IQGAP1. **A** The ASAP-associated interactomes were determined by immunoprecipitation and mass spectrometry (IP-MS). Venn diagrams show the number of proteins that may interact listed in the tables. **B** Correlation analysis of ASAP1 and IQGAP1 RNA expression in TCGA STAD database. **C** Images showing the immunofluorescence of ASAP1 (red) and IQGAP1 (green). Nuclei were counterstained with DAPI (blue). **C** SGC-7901 was transfected with pcDNA3.1 or pcDNA3.1-ASAP1-HA. 24 h after transfection, HA-tag fused proteins were immunoprecipitated with their interacting proteins. Western blot was used to detect indicated proteins. **D** Western blot showing that endogenous ASAP1 co-immunoprecitated with IQGAP1 from SGC-7901 cells. An anti-ASAP1 antibody was used for IP. Normal mouse IgG was used as a control. **E** Western blot showing that HA-tagged ASAP1 coimmunoprecipitated with IQGAP1 from SGC-7901 cells expressing HA-tagged ASAP1, but not from control SGC-7901 cells. An anti-HA antibody was used for IP. **F** Molecular docking pattern diagram of ASAP1 and IQGAP1. **G** IQGAP1 mRNA level of MGC-803 and SGC-7901 after ASAP1 overexpression. **H** IQGAP1 protein level of MGC-803 and SGC-7901 after ASAP1 overexpression. **I** SGC-7901 and SGC-ASAP1^+/−^ cells were incubated with MG132 (+) or DMSO (−) for 4 h. Equal amounts of protein lysates were loaded directly onto gels or immunoprecipitated (IP) with an anti-IQGAP1 antibody. Proteins were analyzed by SDS–PAGE and immunoblotting (IB) using anti-ubiquitin and anti-IQGAP1 antibodies, respectively. **J** Western blot showing that IQGAP was depleted by two different siRNA oligos in SGC-7901 cells. **K** The proliferation of various types of SGC-7901 cells was determined via CCK-8, including cells transfected with pcDNA3.1, cells transfected with pcDNA3.1-ASAP1-HA, cells transfected with pcDNA3,1 followed by transfection with a siRNA oligo against IQGAP1, and cells transfected with pcDNA3.1-ASAP1-HA followed by transfection with a siRNA oligo against IQGAP1. **M** The migration of various types of SGC-7901 cells described in **L** was determined was determined by transwell assays. **N** The who cell lysates were extracted from various types of SGC-7901 cells to measure CDC42 GTPase activity by G-LISA activation assay, including cells transfected with pcDNA3.1, cells transfected with pcDNA3.1-ASAP1-HA, and cells transfected with pcDNA3.1-ASAP1-HA followed by transfection with a siRNA oligo against IQGAP1.

To identify the proteins that interact with ASAP1 in GC cell, we performed immunoprecipitation (IP) with an antibody against ASAP1 followed by label-free mass spectrometric analysis (Fig. 6A). Among the potential ASAP1-interacting proteins, we screened six proteins of interest, including PLEC, RALA, FLNA, RAN, CD55, and IQGAP1. We analyzed the correlation between ASAP1 and the expression levels of these genes using the TCGA-STAD database. The results showed that ASAP1 had the highest correlation with IQGAP1 (R-0.564, *P* < 0.001, Figs. 6B and S3A–E). IQGAP1 is a Ras GTPase-activating-like protein (Ras-GAPs), which can activate GTPases and inactivate the activated Ras proteins. IQGAP also enhances the activity of CDC42. Moreover, IF assay was performed to examine the subcellular distribution of ASAP1 and IQGAP1 in SGC-7901 cells. The results demonstrated that ASAP1 co-localizes with IQGAP1 (Fig. 6C). The co-IP results showed that IQGAP1 was pulled down with either endogenous ASAP1 or HA-tagged ASAP1 protein (Fig. 6D, E). Using LigPlot+ 2.2.4, we found all functional residues and sorted them based on their interactions. We obtained the exact binding energy using Prodigy and found the stability of the two proteins was −13.5 kcal/mol. This was strong evidence for the stability of the protein combination (Fig. 6F)

In addition, we found the IQGAP1 protein level was significantly upregulated after ASAP1 overexpression while the mRNA level did not change significantly (Fig. 6G, H). IQGAP1 is ubiquitinated and its ubiquitination affects CDC42 activation [31]. Therefore, we examined the effects of ASAP1 on IQGAP1 ubiquitination. We treated ASAP1^+/−^ cells with the proteasome inhibitor MG132 to increase ubiquitination of a variety of cellular proteins and detected a single IQGAP1 ubiquitinated band in the IQGAP1 immunoprecipitate with an anti-ubiquitin antibody, whereas there was no obvious band in the control group expressing ASAP1 (Fig. 6I). To determine whether the oncogenic function of ASAP1 depends on IQGAP1, we knocked down IQGAP1 in SGC-7901 cells = and found that the ability of ASAP1 to promote GC cell proliferation and metastasis disappeared (Fig. 6J–M). More importantly, using G-LISA activation assays, we found that the ability of ASAP1 to promote CDC42 activation was eliminated after knockdown of IQGAP1 (Fig. 6N). These results suggest that ASAP1 activates the IQGAP1/CDC42 pathway to promote its oncogenic functions.

### ASAP1 upregulates the EGFR-MAPK pathway and promotes chemotherapy resistance by activating CDC42

**Fig. 7:**
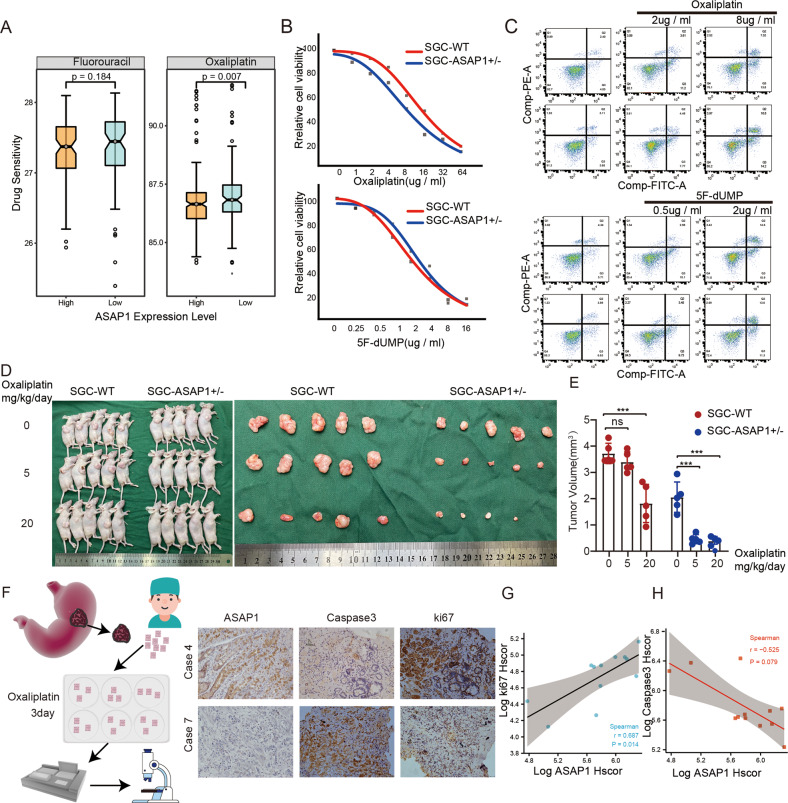
ASAP1 promotes chemotherapy resistance in GC. **A** Drug sensitivity prediction demonstrated that pateints with high expression of ASAP1 tended to be more sensitive to oxaliplatin (*p* = 0.007), whereas there was no statistical difference in the sensitivity to fluorouracil between patients with high and low expression of ASAP1. **B** The effect of ASAP1 on the viability of SGC-7901 cells in response to oxaliplatin or 5-FU. The WT and ASAP1^+/−^ SGC-7901 cells were treated with increasing concentrations of oxaliplatin or 5-FU for 48 h. Cell viability was measured by CCK-8. IC50 values in two different drug treatments are shown (*n* = 3). **C** Flow cytometry analysis showing the comparison of apoptosis induction between WT and ASAP1^+/−^ SGC-7901 cells in response to oxaliplatin. Bar chart showing the percentage of apoptotic cells with mean values ± standard error of the mean (SEM), *n* = 3 biologically independent samples. The data are representative of three experiments with similar results. Statistical analysis was performed using two-tailed unpaired *t*-test. **D**, **E** Subcutaneous tumor model was established and injected different concentrations of Oxa intraperitoneally to nude mice, after 30 days, the nude mice were sacrificed to measure tumor size. **F**–**H** Comparison the activity of gastric cancer tissue 3D culture treated with oxaliplatin. KI67 staining intensity was used to analyze the correlation between ASAP1 expression and tissue viability after oxaliplatin treatment. Caspase3 staining intensity was used to analyze the correlation between ASAP1 expression and apoptosis after oxaliplatin treatment.

5-Fluorouracil (5-FU) and Oxaliplatin (Oxa) are commonly used chemotherapeutic drugs for GC. Drug resistance is a major cause of chemotherapy failure in cancer. We found that, in the TCGA-STAD database, high expression of ASAP1 increased the resistance of GC cells to Oxa, but had no effect on 5-FU resistance (Fig. 7A). The IC50 assay showed that ASAP1^+/−^ SGC-7901 cells were more sensitive than SGC-7901 WT cells to Oxa, but not to 5-FU (Fig. 7B). Flow cytometry was used to detect the effects of the two chemotherapeutic drugs on the apoptosis of ASAP1^+/−^ SGC-7901 cells. The proportion of apoptotic cells in ASAP1^+/−^ cells treated with Oxa was higher than that in the SGC-7901 WT cells, whereas there was no difference between WT and ASAP1^+/−^ SGC-7901 cells in the 5-FU group (Fig. 7C). We injected different concentrations of Oxa intraperitoneally to nude mice inoculated with ASAP1^+/−^ SGC-7901 and SGC-7901 WT subcutaneous tumors. We found that when the Oxa was 5 mg/kg/day, ASAP1^+/−^ SGC-7901 tumor volume was significantly smaller than that in the saline group, while SGC-7901 WT tumor volume was not significantly different from that of the saline group (Fig. 7D, E). To further analyze the relationship between ASAP1 expression in GC tissues and Oxa efficacy, we collected 12 GC tissues for 3D culture with 10 µM Oxa (Fig. S6D). After 3 days of culture, the tissue was fixed and was subjected to immunohistochemical staining for Ki67 and caspase-3. The results showed that caspase-3 expression decreased in tissues with high ASAP1 expression, whereas Ki67 expression increased (Fig. 7F–G). These results indicated that the high ASAP1 expression caused Oxa resistance in patients with GC.

**Fig. 8:**
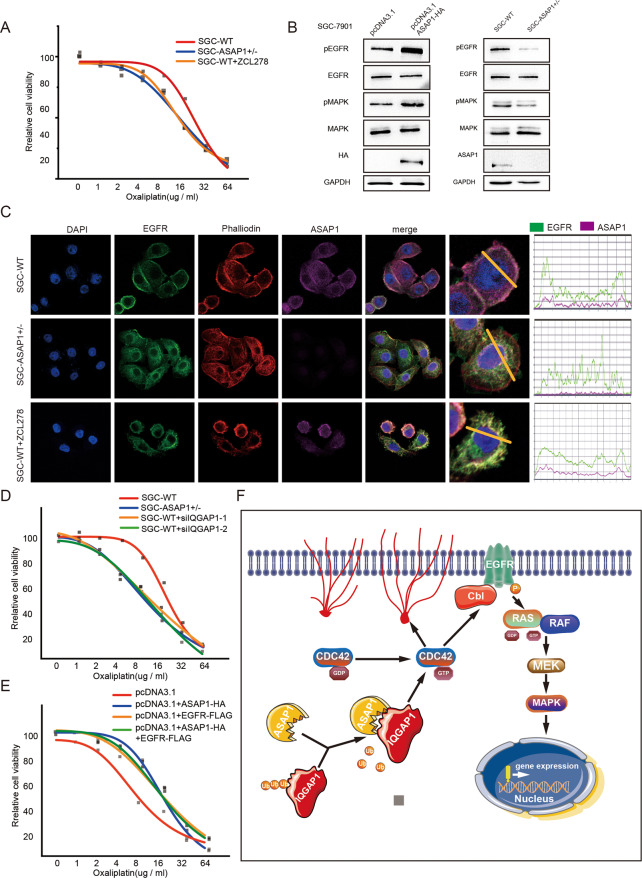
ASAP1 upregulated EGFR-MAPK pathway and promotes chemotherapy resistance by activating CDC42. **A** The effect of ZCL278 on the viability of WT SGC-7901 cells in response to oxaliplatin. Cell viability was measured by CCK-8. IC50 values in two different drug treatments are shown (*n* = 3). **B** WB analysis of EGFR-MAPK pathway in SGC-7901 cells expressing ASAP1-HA and partially knocked out of ASAP1. **C** Phalloidin staining for F-actin (red) and EBFR (green) immunofluorescence. Nuclei were counterstained with DAPI (blue). In ASAP1 ± SGC-7901 cells, the EGFR was not located on the membrane and the cytoskeleton depolymerized. In WT SGC-7901 cells treated with ZCL278, the EGFR was not located on the membrane and the cytoskeleton depolymerized. **D** The effect of IQGAP1 on the viability of SGC-7901-ASAP1 ± cells in response to oxaliplatin. Cell viability was measured by CCK-8. IC50 values in two different drug treatments are shown (*n* = 3). **E** The effect of EGFR on the viability of SGC-7901 cells expressing ASAP1-HA or not in response to oxaliplatin. Cell viability was measured by CCK-8. IC50 values in two different drug treatments are shown (*n* = 3). ns-not significant; *p < 0.05; **p < 0.01; ***p < 0.001. **F** A working model of ASAP1 summarizing this study. ASAP1 was highly expressed in gastric cancer, inhibits ubiquitin-mediated degradation of IQGAP1 and promotes CDC42 activation through interaction with IQGAP1. Activated CDC42 contributes to the growth and metastasis of gastric cancer cells. In addition, activated CDC42 upregulates the EGFR pathway and causes oxaliplatin resistance in gastric cancer.

To analyze whether ASAP1 induced oxaliplatin resistance was related to CDC42 activity. IC50 assay showed that SGC-7901 WT cells treated with ZCL278 eliminated the resistance to Oxa (Fig. 8A). Studies have shown that the carcinogenic EGFR signaling pathway can activate chemotherapy-resistant pathways [32]. RNAactDrug also showed that the level of EGFR expression was negatively correlated with chemotherapeutic drug sensitivity (Fig. S6B, C). In addition, we found the positive correlation between ASAP1 and EGFR expression levels in the TCGA-STAD database (*R* = 0.343, *P* < 0.001, Fig. S6A). CDC42 has been reported to directly engage the EGFR endocytosis machinery for MAPK signaling [33]. Activated CDC42 inhibits the binding of CBI to EGFR, thereby preventing the ubiquitination of EGFR by CBI and increasing the EGFR protein content [34]. To understand whether the mechanism by which ASAP1 enhances drug resistance in tumor cells is related to EGFR, We detected the expression of the EGFR-MAPK pathway in SGC-7901 cells after overexpressing ASAP1 and found that EGFR protein expression, phosphorylated MAPK, and total MAPK expression increased, whereas there was an opposite trend in ASAP1^+/−^-SGC-7901 cells (Fig. 8B) In addition, we investigated the effect of ASAP1 on the subcellular localization of EGFR using an IF assay. We found that EGFR tended to be located on the cell membrane, while EGFR was internalized and degraded in ASAP1^+/−^ cells, resulting in a significant decrease in EGFR on the cell membrane. Cells treated with ZCL278 showed a similar phenomenon (Fig. 8C). IC50 assay showed that SGC-7901 WT cells knockdown of IQGAP1 expression eliminated the resistance to Oxa, and SGC-7901 overexpress EGFR promoted oxaliplatin resistance (Fig. 8D, E). Taken together, ASAP1-activated CDC42 may upregulate the EGFR-MAPK signaling pathway and thus increase the drug resistance in GC cells.

## Discussion

GC is one of the most common malignant gastrointestinal tumors and poses a significant threat to health and life expectancy. Patients with advanced TNM stages usually have an unfavorable prognosis [35]. Since the currently available therapeutic options show limited effectiveness, novel treatment strategies are required to improve clinical outcomes. This is the first systematic exploration of the clinical and prognostic value of ASAP1 in GC by combining bioinformatics analysis with in vitro experiments. ASAP1, a member of the ARF-GAPS family, is involved in membrane-cytoskeleton interactions that affect membrane movements, cell appearance and motility [36]. In our study, endogenous ASAP1 was found to be highly expressed in GC tissues compared to that in normal gastric tissues, and this expression was associated with overall poor survival in patients. ASAP1 overexpression significantly promoted the migration and invasion of GC cells in vitro and promoted the growth of xenograft tumors in vivo. Studies have shown that ASAP1 is associated with tumor development and malignant metastasis in prostate cancer, hepatocellular carcinoma [37], breast cancer [38], head and neck squamous cell carcinomas [39], prostate cancer [40, 41], colorectal cancer [26], and ovarian cancer [25, 42]. Our study shows that high ASAP1 expression promotes migration and invasion; more interestingly, it is associated with the development of EMT in GC cells. Reduced E-cadherin level can lead to reduced cell adhesion, making cells prone to invasion and metastasis. Loss of E-cadherin expression is considered the most significant feature of EMT. Therefore, these results suggest that ASAP1 promotes the epithelial-to-mesenchymal transition and mediates the malignant phenotype of GC.

To elucidate the mechanism by which ASAP1 promotes metastasis, we screened CDC42, a GTPase that interacts with ASAP1 using the G-LISA assay. CDC42, a major member of the Rho-GTPase protein family, controls various cellular processes such as cell migration and morphogenesis through effector factors [43, 44]. To our knowledge, CDC42 is also closely related to tumor invasion, metastasis, and proliferation, and the downregulation of CDC42 can inhibit the migration ability of pancreatic ductal adenocarcinoma cells [45]. The overexpression of CDC42 promotes hepatocellular carcinoma proliferation, metastasis, and tumor growth in vivo [9]. By detecting the cell function of ASAP1 after inhibiting CDC42 with ZCL278, we found that the promoting effect of ASAP1 on proliferation, migration, and invasion disappeared. We confirmed that ASAP1 is dependent on CDC42 to promote GC. Further studies showed that ASAP1 interacts with IQGAP1, which is a regulator of CDC42. By interacting with IQGAP1, CDC42 is stabilized in its active GTP-bound form [46, 47]. IQGAP is localized to the cell–cell contact site, and its overexpression decreases E-cadherin-mediated cell–cell adhesion by interacting with β-catenin and causing the dissociation of α-catenin from the cadherin-catenin complex in epithelial cells [48]. IQGAP1 is a ubiquitinated scaffold protein that interacts with numerous binding partners, thereby regulating fundamental biological processes [49, 50]. ASAP1-induced inhibition of IQGAP1 ubiquitination directly activates CDC42 [51, 52].

In addition, we demonstrated that high ASAP1 expression promotes Oxa resistance in GC cells. The expression of EGFR, an important drug target of Oxa, was negatively correlated with ASAP1 expression. IF assays showed that ASAP1 shifted the subcellular localization of EGFR from the cytoplasm to the cell membrane. The effect of ASAP1 on the subcellular localization of EGFR is mediated by CDC42. CDC42 is an important member of the Rho-GTPase protein family that controls various cellular processes, such as cell migration and morphogenesis, through effector factors [43]. Among these effectors, IQGAP1 plays a pivotal role in the establishment of the cytoskeletal structure and intercellular adhesion of various cells [53, 54]. Ubiquitination of IQGAP1 alters its ability to bind to and activate this GTPase [51]. Studies have shown that the interaction of an activated CDC42 with C-CBL prevents C-CBL from binding to the EGFR, thereby blocking the antagonism to the EGF coupling signal and CBL-catalyzed EGFR ubiquitination [34]. The binding of CDC42 to EGFR is required for EGFR-stimulated receptor-mediated endocytosis and further promotes MAPK signaling [33, 34]. In conclusion, activated CDC42 indirectly acts on CBI, inhibits the ubiquitination of EGFR by CBI, and activates the EGFR-MAPK pathway. As the activation of oncogenic EGFR promotes chemotherapy resistance in GC cells, our findings provide a new target for preventing, controlling, and treating GC metastasis.

In summary, we demonstrated that ASAP1 is upregulated and associated with poor GC prognosis. ASAP1 promotes tumor cell progression and metastasis in vitro. Moreover, ASAP1 was shown to inhibit ubiquitin-mediated degradation of IQGAP1 and directly activates CDC42 (Fig. 8). In addition, we demonstrated that ASAP1 affects platinum-based chemotherapy in GC by activating the EGFR-MAPK signaling pathway. Therefore, the ASAP1-IQGAP1/CDC42 axis may present a new diagnostic and therapeutic target for GC treatment.

## Supplementary information

Change of authorship request

Supplemental table 1

Supplemental table 2

Supplemental table 3

Figure S1

Figure S2

Figure S3

Figure S4

Figure S5

Figure S6

Figure legends for supplemental figure

Original Data File

## Data Availability

All data generated or analyzed during this study are available from the corresponding author on reasonable request.
