# Supplementary material for: ASAP1 activates the IQGAP1/CDC42 pathway to promote tumor progression and chemotherapy resistance in gastric cancer
Source: Cell Death Dis. 2023 Feb 15;14(2):124. doi: 10.1038/s41419-023-05648-9 (PMC9932153; doi:10.1038/s41419-023-05648-9)
Supplement: Supplementary file 1 — Change of authorship request [file 41419_2023_5648_MOESM1_ESM.pdf]

SPRINGER NATURE

Change of authorship request form - Journals (pre-acceptance)

Important information. Please read.

- This form should be used by authors to request any change in authorship (adding/deleting authors) including changes in corresponding authors. This form should not be used for name changes. Please fully complete all sections. Use black ink and block capitals and provide each author's full name with the given name first followed by the family name.
- By signing this declaration, all authors guarantee that the order of the authors are in accordance with their scientific contribution, if applicable as different conventions apply per discipline, and that only authors have been added who made a meaningful contribution to the work.
- Please note, in author collaborations where there is formal agreement for representing the collaboration, it is sufficient for the representative or legal guarantor (usually the corresponding author) to complete and sign the Authorship Change Form on behalf of all authors, **next to the added/removed author(s)**. **(Complete Section 3, followed by Section 6.)** In author collaborations where there is no formal agreement for representing the collaboration and **there are more than 10 authors**, one may sign for all, provided the signer appends correspondence that attests that each of the authors have agreed to the change **and the added/removed authors sign the form**. **(Complete Section 3, followed by Section 6.)**
- Please note, we cannot investigate or mediate any authorship disputes. If you are unable to obtain agreement from all authors (including those who you wish to be removed) you must refer the matter to your institution(s) for investigation. Please inform us if you need to do this.
- If you are not able to return a fully completed form within **30 days** of the date that it was sent to the author requesting the change, we may have to withdraw your manuscript. We cannot publish manuscripts where authorship has not been agreed by all authors (including those who have been removed).
- Incomplete forms will be rejected.
- Please return/upload this form, fully completed, to the Journals Editorial Office. The Journal and/or Publisher will consider the information you have provided to decide whether to approve the proposed change in authorship. We may decide to contact your institution for more information or undertake a further investigation, if appropriate, before making a final decision.

SPRINGER NATURE **Change of authorship request form - Journals** (pre-acceptance)

Section 1: Please provide the current title of manuscript

Manuscript ID no.: CDDIS-22-3331

Title: ASAP1 activates the IQGAP1/CDC42 pathway to promote tumor progression and chemotherapy resistance in gastric cancer

Section 2: Please provide the previous authorship, in the order shown on the manuscript before the changes were introduced. Please indicate the corresponding author by adding (CA) behind the name.

	First name(s)	Family name	ORCID or SCOPUS id, if available
1st author	Wangkai	Xie	
2nd author	Zheng	Han	
3rd author	Ziyi	Zuo	
4th author	Hua	Chen	
5th author	Juanjuan	Huang	
6th author	Siyu	Zhu	
7th author	Han	Lou	
8th author	Zhiqiang	Yu	
9th author	Chenbin	Chen	
10th author	Dong	Xin	
11th author	Sian	Chen	
12th author	Yuanbo	Hu	
13th author	Jingjing	Huang	
14th author	Fabiao	Zhang	
15th author	Xian	Shen	
16th author	Xiangyang	Xue	
17th author	Kezhi	Lin	

Please use an additional sheet if there are more than 10 authors.

SPRINGER NATURE

Change of authorship request form - Journals (pre-acceptance)

Section 3: Please provide a justification for change. Please use this section to explain your reasons for changing the authorship of your manuscript, e.g. what necessitated the change in authorship? Please refer to the (journal) policy pages for more information about authorship. Please explain why omitted authors were not originally included and/or why authors were removed on the submitted manuscript.

In the article revision stage, Dr Dong Xin made important contributions by completing the animal experiments and collating the data, he is the 10th author in previous authorship and is now applying to list him as a co-first author.

Dr. Zonglin Ni made important contributions in the revision stage of the article and now applies to list him as an author.

Section 4: Proposed new authorship. Please provide your new authorship list in the order you would like it to appear on the manuscript. Please indicate the corresponding author by adding (CA) behind the name. <u>If the Corresponding Author has changed, please indicate the reason under section 3</u>.

	First name(s)	Family name (this name will appear in full on the final publication and will be searchable in various abstract and indexing databases)	Affiliated institute	E-mail address
1st author	Wangkai	Xie	Department of General Surgery, The Second Affiliated Hospital and Yuying Children's Hospital of Wenzhou Medical University	757774124@qq.com
2nd author	Zheng	Han	Department of General Surgery, The Second Affiliated Hospital and Yuying Children's Hospital of Wenzhou Medical University	hz_1213@qq.com
3rd author	Ziyi	Zuo	Department of General Surgery, The First Affiliated Hospital of Wenzhou Medical University	jerryzuo0214@msn.cn
4th author	Dong	Xin	Wenzhou Collaborative Innovation Center of Gastrointestinal Cancer in Basic Research and Precision Medicine	1063259676@qq.com
5th author	Hua	Chen	Department of General Surgery, The First Affiliated Hospital of Wenzhou Medical University	1063259676@qq.com
6th author	Juanjuan	Huang	Wenzhou Collaborative Innovation Center of Gastrointestinal Cancer in Basic Research and Precision Medicine	467565546@qq.com
7th author	Siyu	Zhu	Wenzhou Collaborative Innovation Center of Gastrointestinal Cancer in Basic Research and Precision Medicine	958112364@qq.com
8th author	Han	Lou	Wenzhou Collaborative Innovation Center of Gastrointestinal Cancer in Basic Research and Precision Medicine	624717107@qq.com

SPRINGER NATURE Springer Nature is one of the world's leading global research, educational and professional publishers, created in May 2015 through the combination of Nature Publishing Group, Palgrave Macmillan, Macmillan Education and Springer Science+Business Media.

SPRINGER NATURE

Change of authorship request form - Journals (pre-acceptance)

	First name(s)	Family name (this name will appear in full on the final publication and will be searchable in various abstract and indexing databases)	Affiliated institute	E-mail address
9th author	Zhiqiang	Yu	Wenzhou Collaborative Innovation Center of Gastrointestinal Cancer in Basic Research and Precision Medicine	1169270810@qq.com
10th author	Chenbin	Chen	Department of General Surgery, The Second Affiliated Hospital and Yuying Children's Hospital of Wenzhou Medical University	1551156168@qq.com
11th author	Sian	Chen	Department of emergency, The Second Affiliated Hospital and Yuying Children's Hospital of Wenzhou Medical University	136105041@qq.com
12th author	Yuanbo	Hu	Department of General Surgery, The Second Affiliated Hospital and Yuying Children's Hospital of Wenzhou Medical University	732267575@qq.com
13th author	Jingjing	Huang	Department of Pathology, The Second Affiliated Hospital and Yuying Children's Hospital of Wenzhou Medical University	maoer0923@163.com
14th author	Fabiao	Zhang	Key Laboratory of Minimally Invasive Techniques & Rapid Rehabilitation of Digestive System Tumor of Zhejiang Province	784671568@qq.com
15th author	Zonglin	Ni	Department of General Surgery, The Second Affiliated Hospital and Yuying Children's Hospital of Wenzhou Medical University	17357571887@163.com
16th author	Xian	Shen	Department of General Surgery, The Second Affiliated Hospital and Yuying Children's Hospital of Wenzhou Medical University	13968888872@163.com
17th author	Xiangyang	Xue	Wenzhou Collaborative Innovation Center of Gastrointestinal Cancer in Basic Research and Precision Medicine	wzxxy001@163.com
18th author	Kezhi	Lin	Wenzhou Collaborative Innovation Center of Gastrointestinal Cancer in Basic Research and Precision Medicine	lkz@wmu.edu.cn

SPRINGER NATURE Springer Nature is one of the world's leading global research, educational and professional publishers, created in May 2015 through the combination of Nature Publishing Group, Palgrave Macmillan, Macmillan Education and Springer Science+Business Media.

SPRINGER NATURE

Change of authorship request form - Journals (pre-acceptance)

Section 5: Author contribution, Acknowledgement and Disclosures. Please use this section to provide a new disclosure statement and, if appropriate, acknowledge any contributors who have been removed as authors and ensure you state what contribution any new authors made (if applicable per the journal or book (series) policy). Please ensure these are updated in your manuscript - <u>after approval of the change(s)</u> - as our production department will not transfer the information in this form to your manuscript.

New acknowledgements:

We thank all patients who participated in this study and everyone who contributed to study.

New Disclosures (financial and non-financial interests, funding):

The authors declare that there is no conflict of interests.

This study was supported by grants from the National Natural Science Foundation of China (Grant Nos: 32070151), the Leading Talents in Scientific and Technological Innovation from Zhejiang Provincial Ten Thousand Talents Plan (Grant Nos: 2019R52021), and the Key R&D Program of Zhejiang Province (Grant Nos: 2020C03029, 2021C03120), the Zhejiang Provincial Natural Science Foundation of China (Grant No. LY19H190005)

New Author Contributions statement (if applicable per the journal policy):

Study conception and design, X.S., X.X., K.L.; experiment, W.X., Z.H., J.H., D.X., S.Z.; analyze bioinformatics data, Z.Z., H.C.; data collection, H.L., C.C., S.C.,Y.H., H.J., F.Z., Z.N.; writing and original draft preparation, W.X

State 'Not applicable' if there are no new authors.

SPRINGER NATURE Springer Nature is one of the world's leading global research, educational and professional publishers, created in May 2015 through the combination of Nature Publishing Group, Palgrave Macmillan, Macmillan Education and Springer Science+Business Media.

SPRINGER NATURE

Change of authorship request form - Journals (pre-acceptance)

Section 6: Declaration of agreement. *All* authors, unchanged, new and removed *must* sign this declaration.
(NB: Please print the form, (docu)-sign and return/upload a scanned copy. Please note that signatures that have been inserted as an image file are acceptable as long as it is handwritten. Typed names in the signature box are unacceptable.) * Please delete as appropriate. **Delete all of the bold if you were on the original authorship list and are remaining as an author.**

	First name	Family name		Signature	Date
1st author	Wangkai	Xie	I agree to the proposed new authorship shown in section 4 **/and the addition/removal* of my name to the authorship list** /and the proposed change in corresponding author	XieWangkai	
2nd author	Zheng	Han	I agree to the proposed new authorship shown in section 4 **/and the addition/removal* of my name to the authorship list** /and the proposed change in corresponding author	Han Zheng	
3rd author	Ziyi	Zuo	I agree to the proposed new authorship shown in section 4 **/and the addition/removal* of my name to the authorship list** /and the proposed change in corresponding author	ZiYi Zuo	
4th author	Dong	Xin	I agree to the proposed new authorship shown in section 4 **/and the addition/removal* of my name to the authorship list** /and the proposed change in corresponding author	Dong Xin	
5th author	Hua	Chen	I agree to the proposed new authorship shown in section 4 **/and the addition/removal* of my name to the authorship list** /and the proposed change in corresponding author	CHen Hua	
6th author	Juanjuan	Huang	I agree to the proposed new authorship shown in section 4 **/and the addition/removal* of my name to the authorship list** /and the proposed change in corresponding author	[illegible]	
7th author	Siyu	Zhu	I agree to the proposed new authorship shown in section 4 **/and the addition/removal* of my name to the authorship list** /and the proposed change in corresponding author	zhu si Yu	
8th author	Han	Lou	I agree to the proposed new authorship shown in section 4 **/and the addition/removal* of my name to the authorship list** /and the proposed change in corresponding author	Han Lou	
9th author	Zhiqiang	Yu	I agree to the proposed new authorship shown in section 4 **/and the addition/removal* of my name to the authorship list** /and the proposed change in corresponding author	Yu Zhiqiang	

SPRINGER NATURE

Change of authorship request form - Journals (pre-acceptance)

	First name	Family name		Signature	Date
10 author	Chenbin	Chen	I agree to the proposed new authorship shown in section 4 **/and the addition/removal* of my name to the authorship** list /and the proposed change in corresponding author	[illegible] Chen Chenbin	
11th author	Sian	Chen	I agree to the proposed new authorship shown in section 4 **/and the addition/removal* of my name to the authorship** list /and the proposed change in corresponding author	陈斯安 Chen Sian	
12th author	Yuanbo	Hu	I agree to the proposed new authorship shown in section 4 **/and the addition/removal* of my name to the authorship** list /and the proposed change in corresponding author	胡渊博 Hu Yuanbo	
13th author	Jingjing	Huang	I agree to the proposed new authorship shown in section 4 **/and the addition/removal* of my name to the authorship** list /and the proposed change in corresponding author	黄京京 Jingjing Huang	
14th author	Fabiao	Zhang	I agree to the proposed new authorship shown in section 4 **/and the addition/removal* of my name to the authorship** list /and the proposed change in corresponding author	[illegible] Fabiao Zhang	
15th author	Zonglin	Ni	I agree to the proposed new authorship shown in section 4 **/and the addition/removal* of my name to the authorship** list /and the proposed change in corresponding author	倪仲林 Ni zhong Lin	
16th author	Xian	Shen	I agree to the proposed new authorship shown in section 4 **/and the addition/removal* of my name to the authorship** list /and the proposed change in corresponding author	沈贤 Xian Shen	
17th author	Xiangyang	Xue	I agree to the proposed new authorship shown in section 4 **/and the addition/removal* of my name to the authorship** list /and the proposed change in corresponding author	薛向阳 Xiangyang Xue	
18th author	Kezhi	Lin	I agree to the proposed new authorship shown in section 4 **/and the addition/removal* of my name to the authorship** list /and the proposed change in corresponding author	[illegible] Kezhi Lin	

Please use an additional sheet if there are more than 10 authors.

SPRINGER NATURE Change of authorship request form - Journals (pre-acceptance)

In case of author collaborations with formal agreement:

	Name of consortium/ consortia	First name	Family name		Signature	Date
Representative/ legal guarantor				I agree to the proposed new authorship shown in section 4 /**and the addition/ removal* of my name to the authorship list** /and the proposed change in corresponding author		

Both added/ removed authors should complete the information in the first table under Section 6.

---- End of form ----
