# Supplementary material for: ASAP1 activates the IQGAP1/CDC42 pathway to promote tumor progression and chemotherapy resistance in gastric cancer
Source: Cell Death Dis. 2023 Feb 15;14(2):124. doi: 10.1038/s41419-023-05648-9 (PMC9932153; doi:10.1038/s41419-023-05648-9)
Supplement: Supplementary file 2 — Supplemental table 1 [file 41419_2023_5648_MOESM2_ESM.docx]

**Table S1.** Primer sequences

| **Primer** | | **Nucleotide Sequence** |
| --- | --- | --- |
| *ASAP1* | Forward | 5’-TAGAACAGCCCTTCAGAAAGTGA-3’ |
|  | Reverse | 5’-CGGGGTTGTCTCGACTTAAAAA-3’ |
| *GAPDH* | Forward | 5’-CAGGGCTGCTTTTAACTCTGGTAA-3’ |
|  | Reverse | 5’-GGGTGGAATCATATTGGAACATGT-3’ |
