# Supplementary material for: ASAP1 activates the IQGAP1/CDC42 pathway to promote tumor progression and chemotherapy resistance in gastric cancer
Source: Cell Death Dis. 2023 Feb 15;14(2):124. doi: 10.1038/s41419-023-05648-9 (PMC9932153; doi:10.1038/s41419-023-05648-9)
Supplement: Supplementary file 3 — Supplemental table 2 [file 41419_2023_5648_MOESM3_ESM.docx]

**Table S2.** siRNA sequences

| **siRNA** | | **Nucleotide Sequence** |
| --- | --- | --- |
| *ASAP1* | si-1 | 5’- CCCAAAUUGGAGAUUUGCCGCCUAA -3’ |
|  | si-2 | 5’- GACCAGAUCUCUGUCUCGGAGUUCA -3’ |
| *IQGAP1* | si-1 | 5’-GAACGTGGCTTATGAGTAC-3’ |
|  | si-2 | 5’-GGCATATCAAGATCGGTTA-3’ |
