# Supplementary material for: ASAP1 activates the IQGAP1/CDC42 pathway to promote tumor progression and chemotherapy resistance in gastric cancer
Source: Cell Death Dis. 2023 Feb 15;14(2):124. doi: 10.1038/s41419-023-05648-9 (PMC9932153; doi:10.1038/s41419-023-05648-9)
Supplement: Supplementary file 4 — Supplemental table 3 [file 41419_2023_5648_MOESM4_ESM.docx]

**Table** S3. List of antibodies used in the study

| Antibodies | Dilution | Source | Address |
| --- | --- | --- | --- |
| Goat anti-Rabbit IgG (H+L) Highly Cross-Adsorbed Secondary Antibody, Alexa Fluor™ 488 | 1:1000 IF | A-11034, Invitrogen | Grand Island, NY, USA |
| Goat anti-Mouse IgG (H+L) Cross-Adsorbed Secondary Antibody, Alexa Fluor™ 647 | 1:500 IF | A-21235, Invitrogen | Grand Island, NY, USA |
| Alexa Fluor™ 594 Phalloidin | 1:50 IF | A12381, Invitrogen | Grand Island, NY, USA |
| IgG | 5ug IP | sc-2343, Santa cruz | Santa Cruz, CA, USA |
| Goat Anti-Rabbit IgG H&L (HRP) | 1:5000 WB | AB97051, ABCAM | Cambridge, MA, USA |
| Goat Anti-Mouse IgG H&L (HRP) | 1:5000 WB | AB6789, ABCAM | Cambridge, MA, USA |
| ASAP1 | 1:50 IHC  1:50 IF  1:200 WB  5ug IP | sc-374410, Santa cruz | Santa Cruz, CA, USA |
| HA-Tag | 1:1000 WB  1:1000 IF | 3724, Cell Signaling Technology | Danvers, MA, USA |
| GAPDH | 1:1000 WB | 5174, Cell Signaling Technology | Danvers, MA, USA |
| IQGAP1 | 1:2000 WB  4ug IP | 22167-1-AP, Proteintech | Rosemont, PA, USA |
| Ubiquitin | 1:1000 WB | 3936, Cell Signaling Technology | Danvers, MA, USA |
| CDC42 | 1:200 WB, | sc-8401, Santa cruz | Santa Cruz, CA, USA |
| EGFR | 1:1000 WB | 2085, Cell Signaling Technology | Danvers, MA, USA |
| p-EGFR | 1:1000 WB | 3777, Cell Signaling Technology | Danvers, MA, USA |
| MAPK | 1:1000 WB | 9102, Cell Signaling Technology | Danvers, MA, USA |
| p-MAPK | 1:1000 WB | 4377, Cell Signaling Technology | Danvers, MA, USA |
| Ki67 | 1:1000 IHC | 9449, Cell Signaling Technology | Danvers, MA, USA |
| Caspase 3 | 1:1000 IHC | 9662, Cell Signaling Technology | Danvers, MA, USA |
