# Supplementary figures and images for: ASAP1 activates the IQGAP1/CDC42 pathway to promote tumor progression and chemotherapy resistance in gastric cancer
Source: Cell Death Dis. 2023 Feb 15;14(2):124. doi: 10.1038/s41419-023-05648-9 (PMC9932153; doi:10.1038/s41419-023-05648-9)

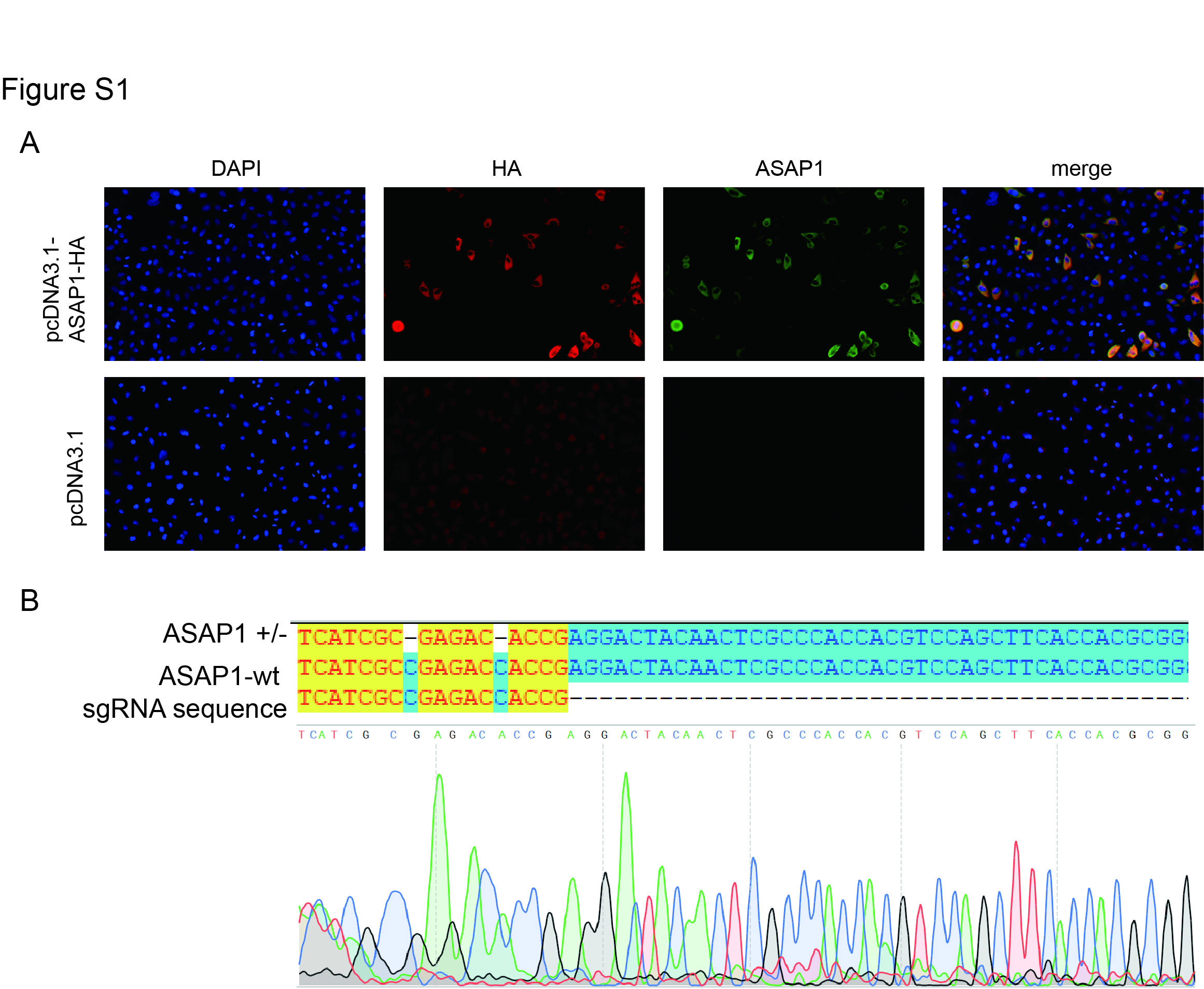

Supplement: Supplementary file 5 — Figure S1 [file 41419_2023_5648_MOESM5_ESM.tif]

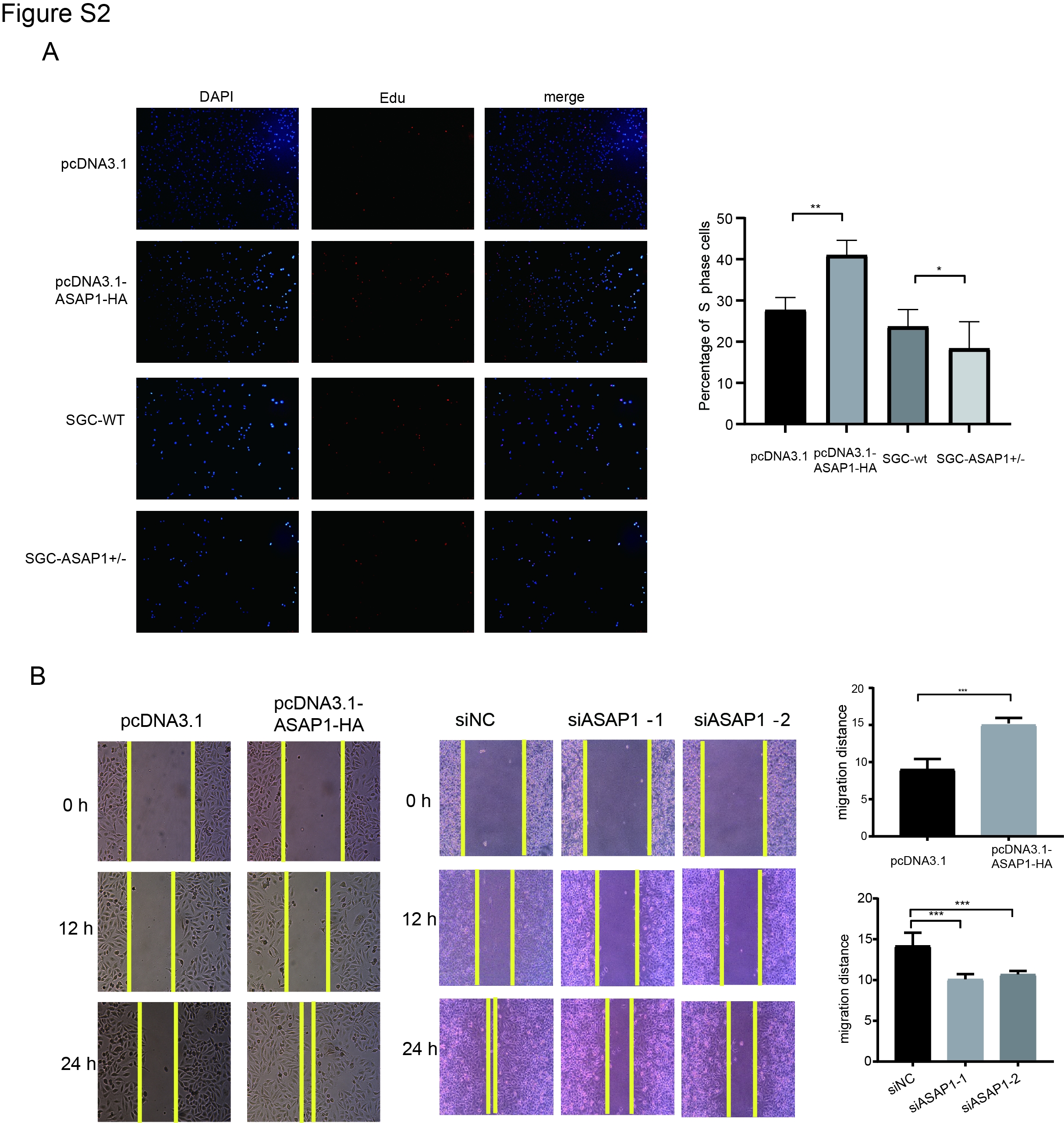

Supplement: Supplementary file 6 — Figure S2 [file 41419_2023_5648_MOESM6_ESM.tif]

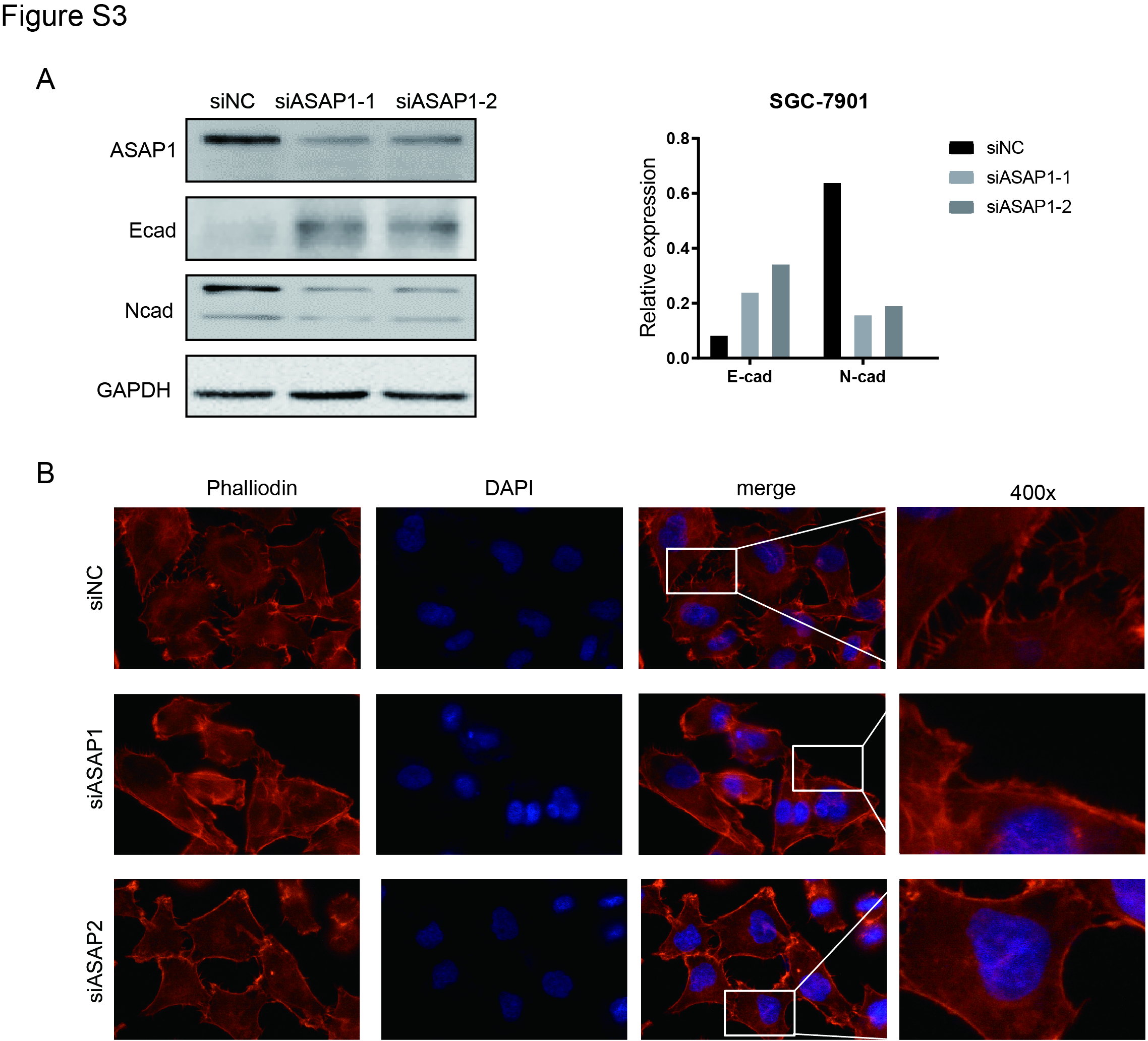

Supplement: Supplementary file 7 — Figure S3 [file 41419_2023_5648_MOESM7_ESM.tif]

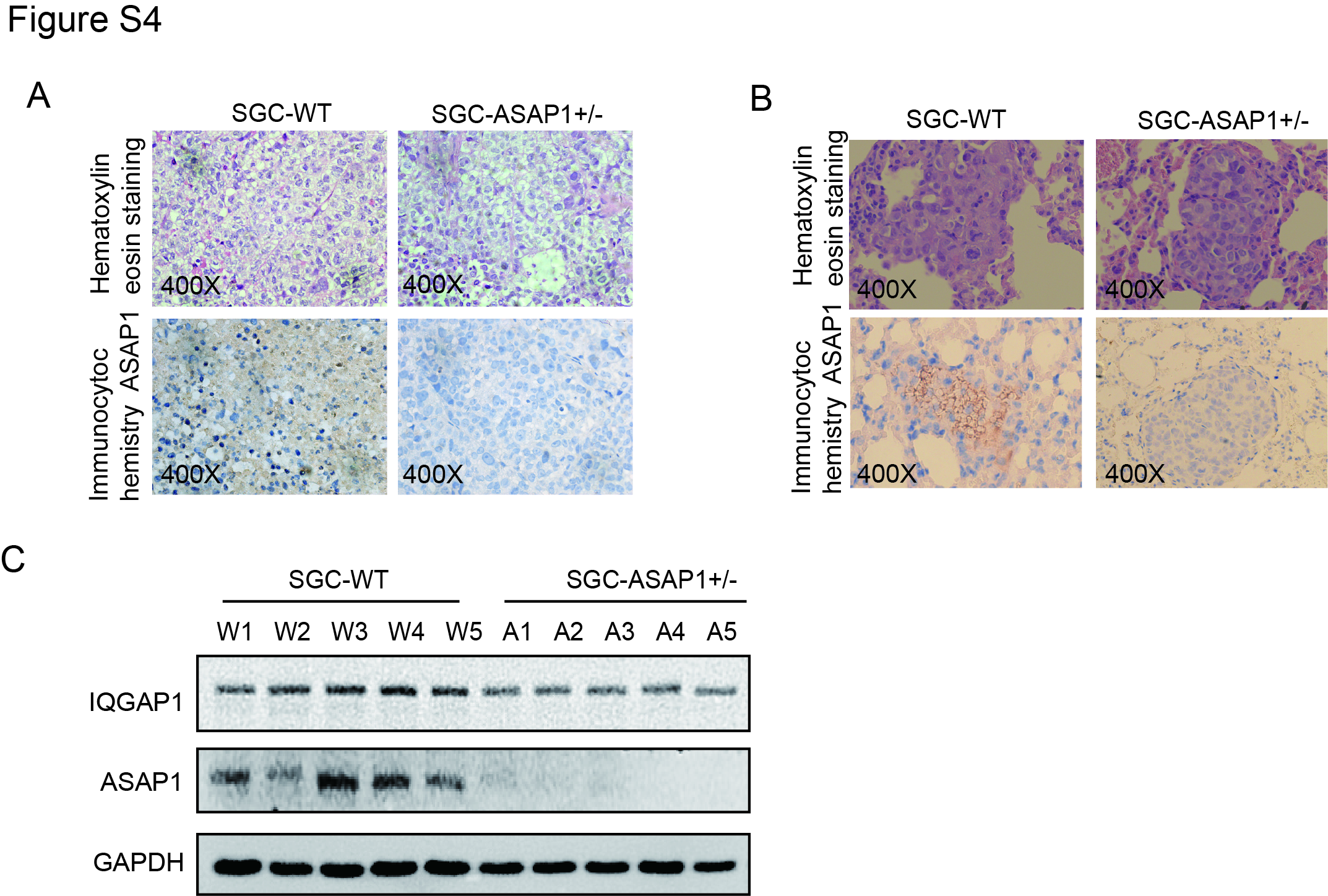

Supplement: Supplementary file 8 — Figure S4 [file 41419_2023_5648_MOESM8_ESM.tif]

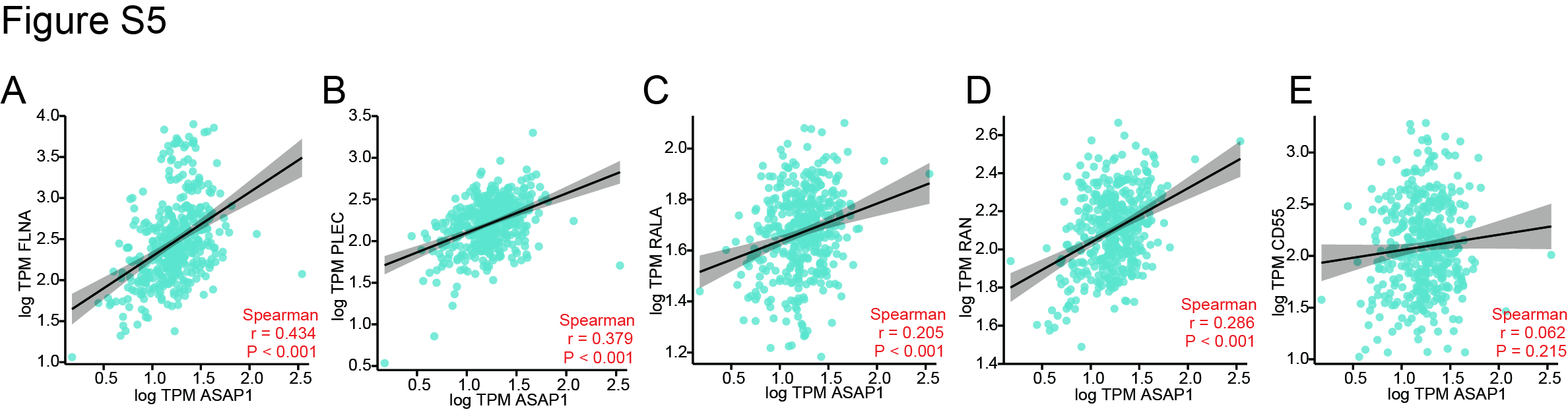

Supplement: Supplementary file 9 — Figure S5 [file 41419_2023_5648_MOESM9_ESM.tif]

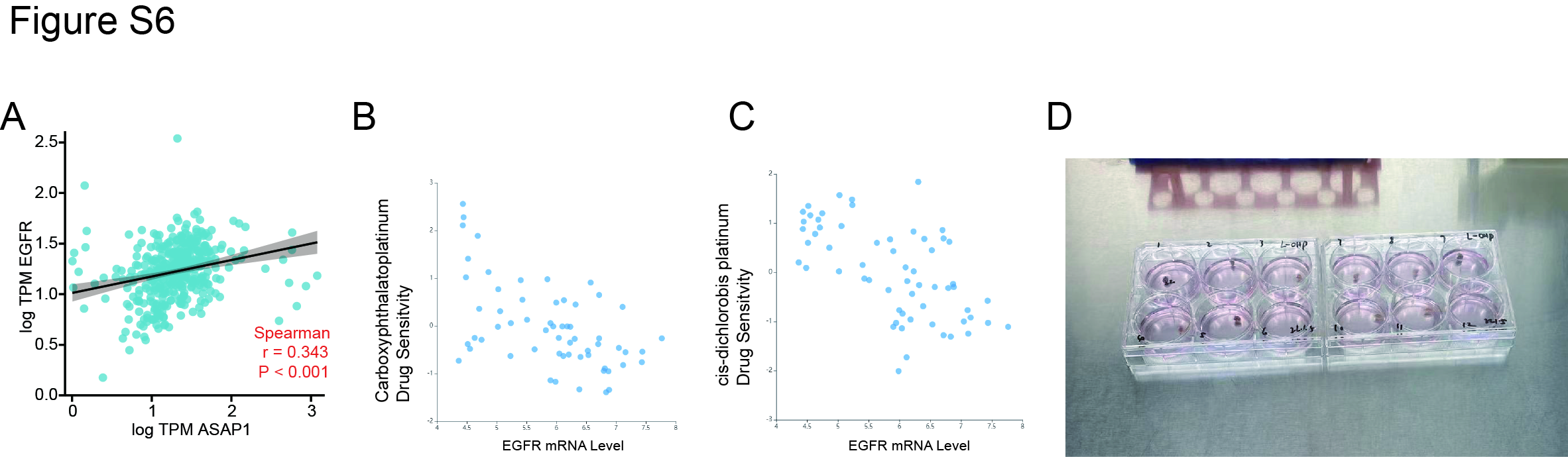

Supplement: Supplementary file 10 — Figure S6 [file 41419_2023_5648_MOESM10_ESM.tif]
