# Supplementary material for: ASAP1 activates the IQGAP1/CDC42 pathway to promote tumor progression and chemotherapy resistance in gastric cancer
Source: Cell Death Dis. 2023 Feb 15;14(2):124. doi: 10.1038/s41419-023-05648-9 (PMC9932153; doi:10.1038/s41419-023-05648-9)
Supplement: Supplementary file 11 — Figure legends for supplemental figure [file 41419_2023_5648_MOESM11_ESM.docx]

**Figure S1**

(A) Gastric cancer cells were transfected with ASAP1 plasmid and incubated with anti-ASAP1(red) and anti-HA (green) by immunofluorescence. HA and ASAP1 co-locate in cells. (B) Genomic sequencing analysis of SGC-7091 partial knockout cells.

**Figure S2**

(A) The proliferation of SGC-7901 cells expressing ASAP1-HA or partially knocked out of ASAP1 was determined via EdU assays. (B) The migration capacity of SGC-7901 cells expressing ASAP1-HA or partially knocked out of ASAP1 by CRISPR-Cas9 was determined via Wound scratch assay. Scale bar,100 μm. *P < 0.05, **P < 0.01, and ***P < 0.001.

**Figure S3**

(A) Western blotting showed that down-regulating the expression level of ASAP1 the expression of Ncad decreased and the expression of E-cad up-regulated in SGC-7901 cells. (B) si-ASAP1 was transfected into SGC-7901, the cytoskeleton was labeled with cytoskeleton was labeled with Phalloidin.

**Figure S4**

（A）Hematoxylin-eosin (HE) staining and immunohistochemistry of the subcutaneous tumor tissue. (B) Hematoxylin-eosin (HE) staining and immunohistochemistry of the tumors with metastasis. (C) Western blotting showed the expression level of ASAP1 and IQGAP1 of subcutaneous tumor tissue.

**Figure S5**

(A-E) The correlation between the expression levels of ASAP1 and PLEC, RALA, FLNA, RAN, CD55 in TCGA-STAD database.

**Figure S6**

(A) The correlation between the expression levels of ASAP1 and EGFR in TCGA-STAD database. (B, C) RNAactDrug was used to analyze the relationship between EGFR expression and platinum sensitivity. (D) Image showing culture of human gastric cancers in 3D culture system.
