# Supplementary material for: ASAP1 activates the IQGAP1/CDC42 pathway to promote tumor progression and chemotherapy resistance in gastric cancer
Source: Cell Death Dis. 2023 Feb 15;14(2):124. doi: 10.1038/s41419-023-05648-9 (PMC9932153; doi:10.1038/s41419-023-05648-9)
Supplement: Supplementary file 12 — Original Data File [file 41419_2023_5648_MOESM12_ESM.pdf]

Full and uncropped western blot for Figure3A

ASAP1

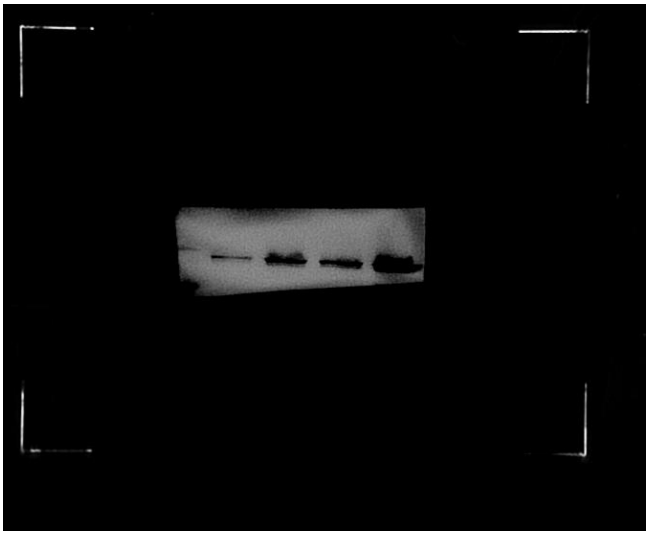

132kDa

HA

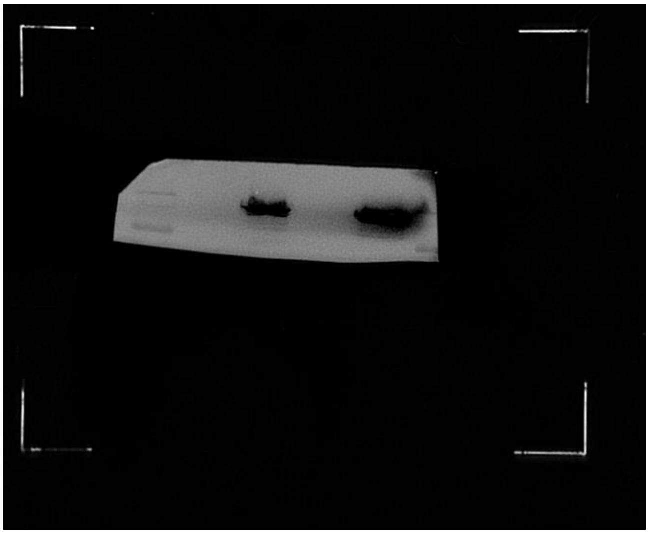

132kDa

GAPDH

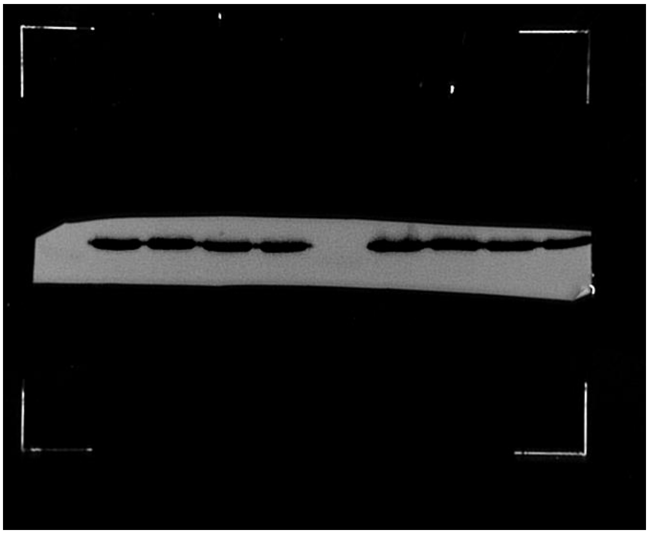

37kDa

Full and uncropped western blot for Figure3D

ASAP1

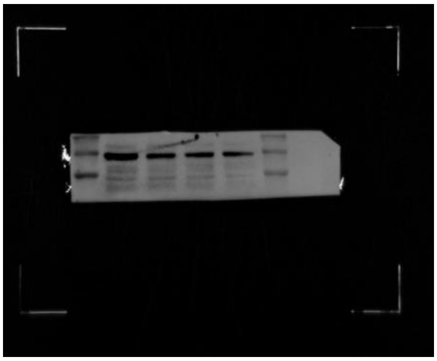

132kDa

GAPDH

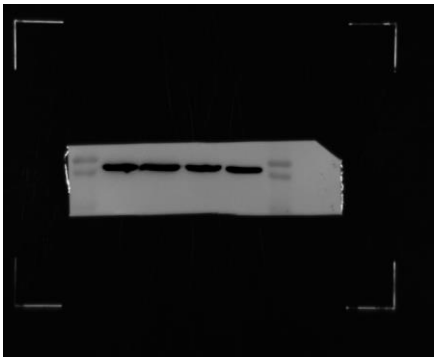

37kDa

ASAP1

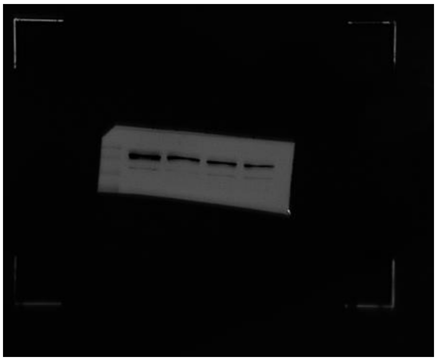

132kDa

GAPDH

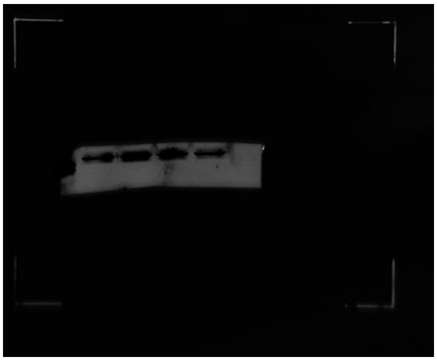

37kDa

Full and uncropped western blot for Figure3G

ASAP1

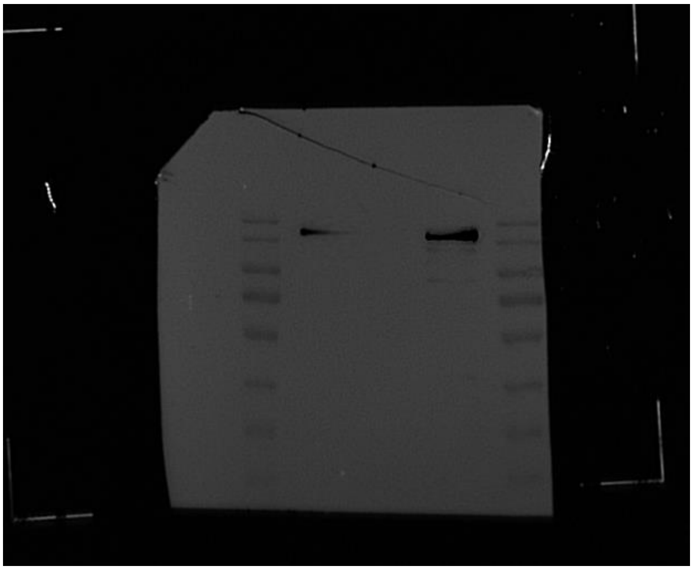

132kDa

GAPDH

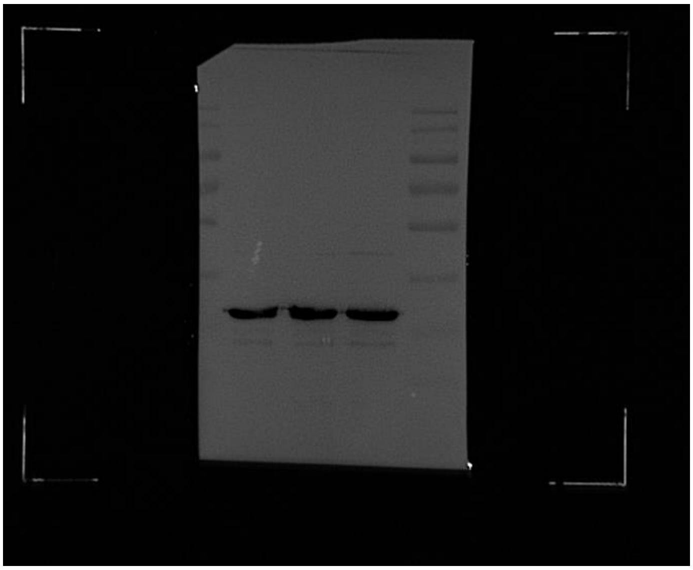

37kDa

Full and uncropped western blot for Figure5D

ASAP1

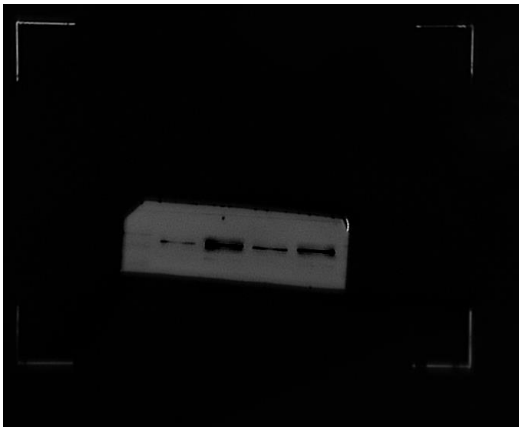

132kDa

CDC42

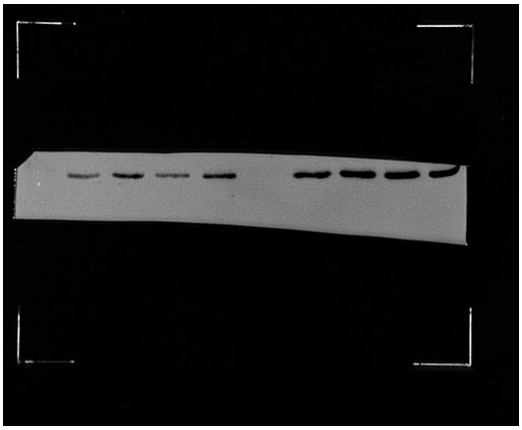

21kDa

GAPDH

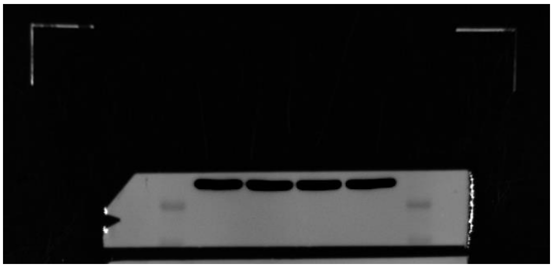

37kDa

Full and uncropped western blot for Figure6

IQGAP1

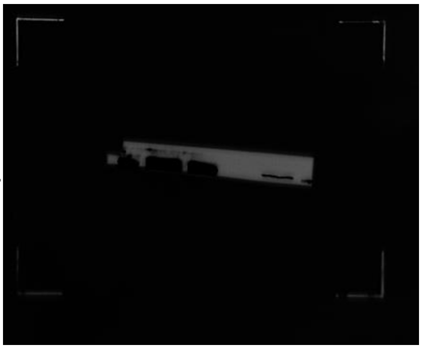

190kDa

IQGAP1

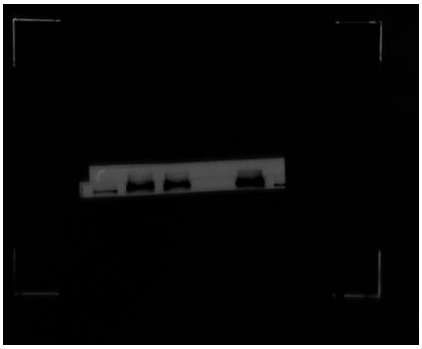

190kDa

ASAP1

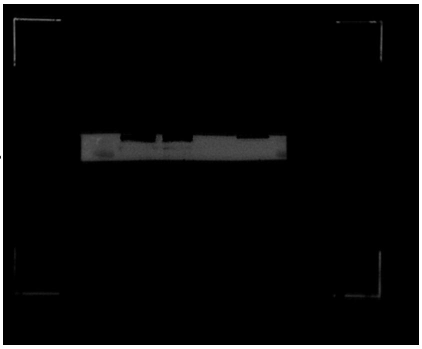

132kDa

HA

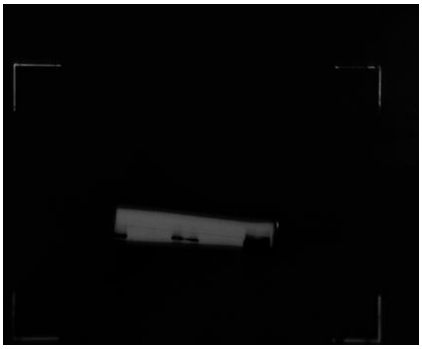

132kDa

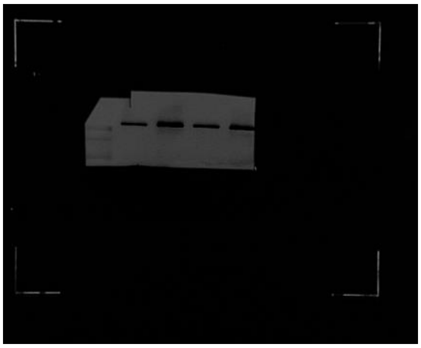

IQGAP1 190kDa

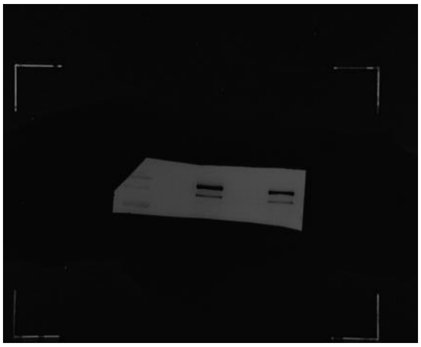

HA 132kDa

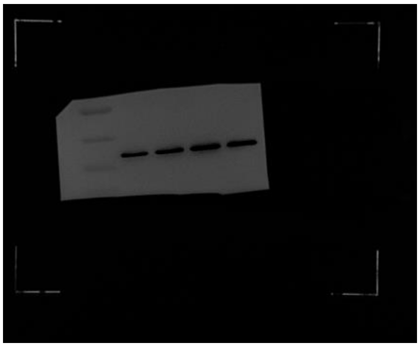

GAPDH 37kDa

Ub

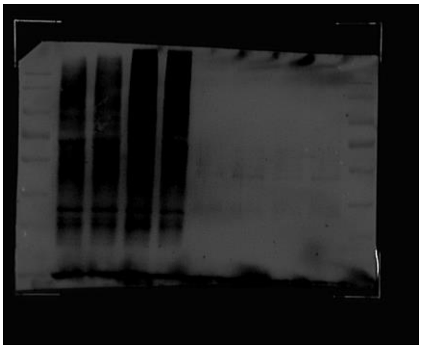

190kDa

IQGAP1

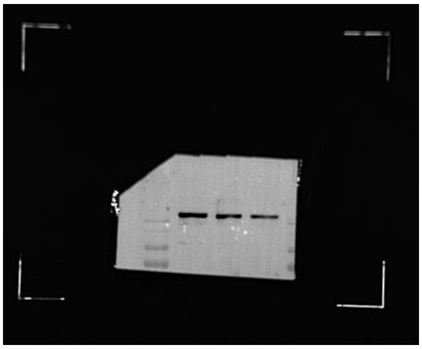

190kDa

IQGAP1

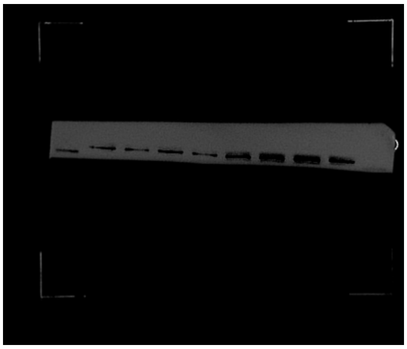

190kDa

GAPDH

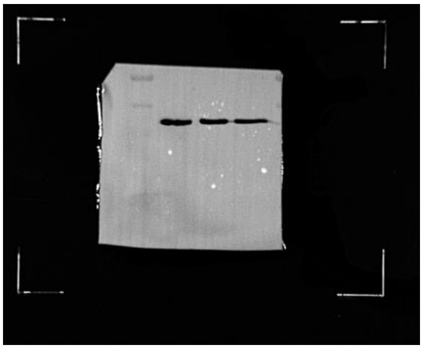

37kDa

Full and uncropped western blot for Figure7

HA

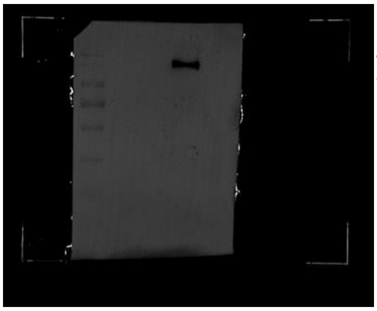

132kDa

ASAP1

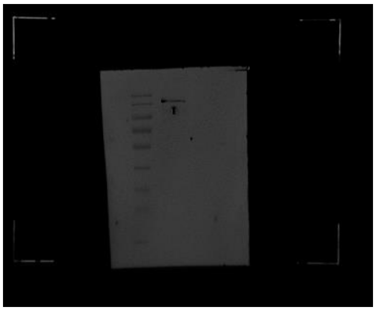

132kDa

EGFR

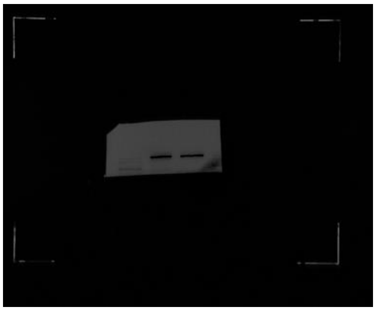

134kDa

EGFR

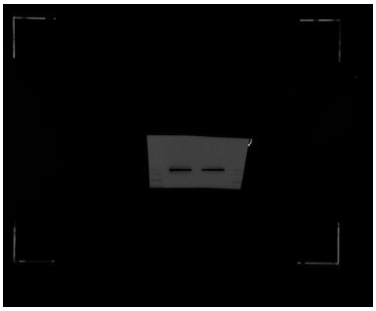

134kDa

PEGFR

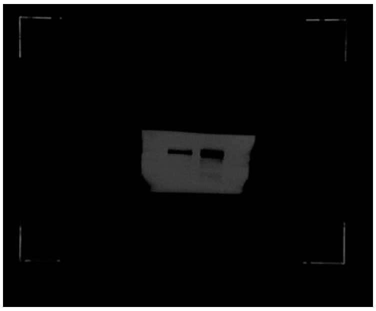

134kDa

PEGFR

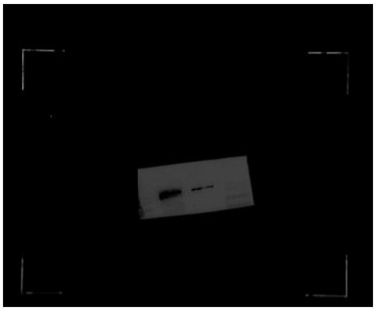

134kDa

MAPK

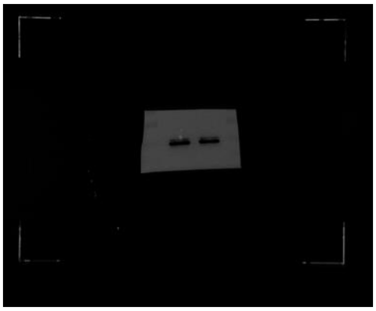

40/42kDa

MAPK

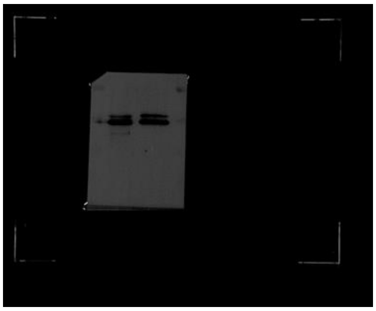

40/42 kDa

pMAPK

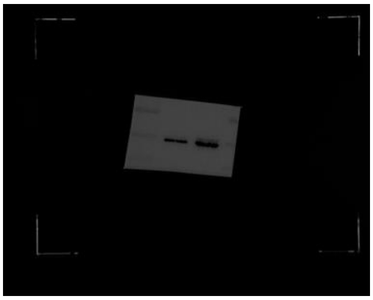

40/42kDa

pMAPK

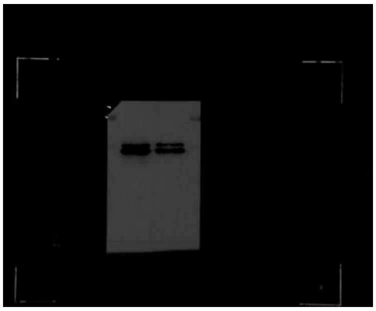

40/42 kDa

GAPDH

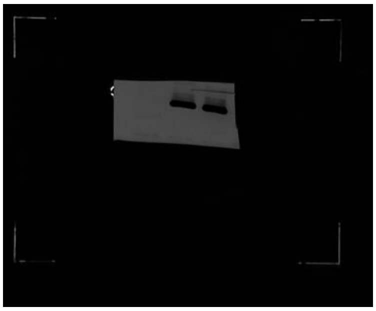

37kDa

GAPDH

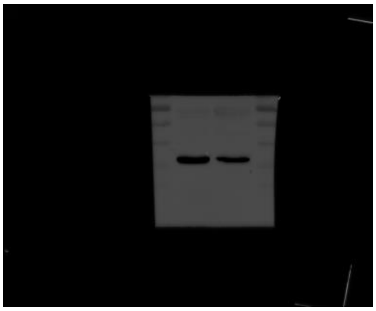

37kDa

Full and uncropped western blot for FigureS3

ASAP1

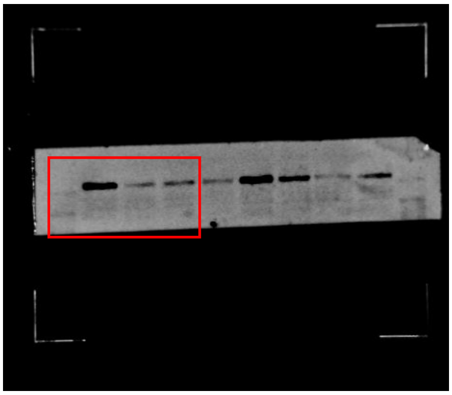

132kDa

Ecad

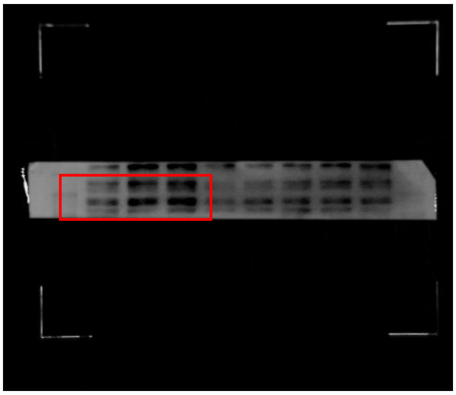

125kDa

Ncad

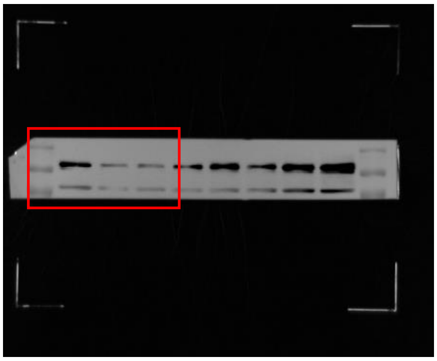

130kDa

GAPDH

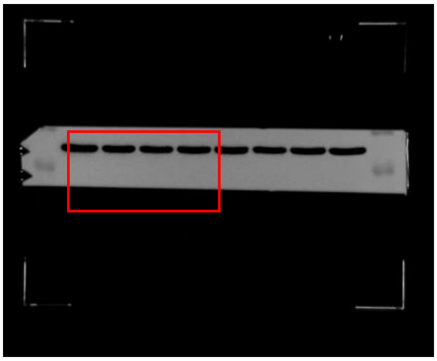

37kDa

Full and uncropped western blot for FigureS4

IQGAP1

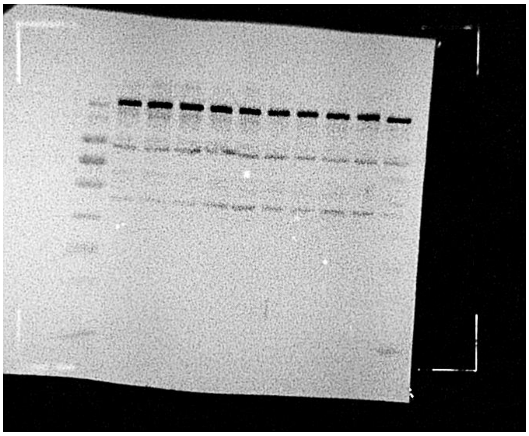

190kDa

GAPDH

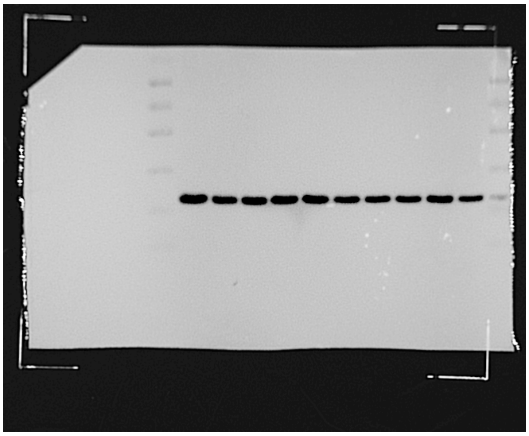

37kDa

ASAP1

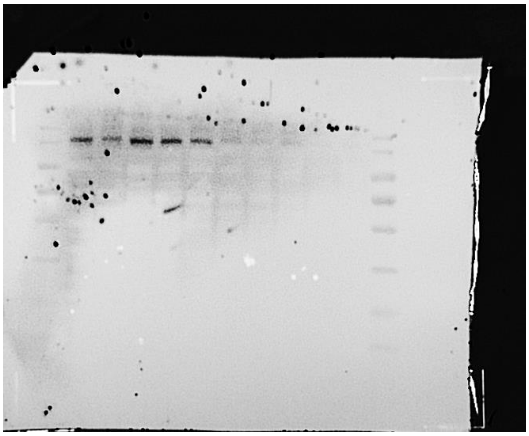

132kDa
